# The genome of the miiuy croaker reveals well-developed innate immune and sensory systems

**DOI:** 10.1038/srep21902

**Published:** 2016-02-23

**Authors:** Tianjun Xu, Guoliang Xu, Rongbo Che, Rixin Wang, Yanjin Wang, Jinrui Li, Shanchen Wang, Chang Shu, Yuena Sun, Tianxing Liu, Jiang Liu, Aishuai Wang, Jingjing Han, Qing Chu, Qiong Yang

**Affiliations:** 1 Laboratory of Fish Biogenetics & Immune Evolution, College of Marine Science, Zhejiang Ocean University, Zhoushan, 316022, China

## Abstract

The miiuy croaker, *Miichthys miiuy*, is a representative Sciaenidae known for its exceptionally large otoliths. This species mainly inhabits turbid aquatic environments with mud to sandy mud bottoms. However, the characteristics of the immune system of this organism and its specific aquatic environment adaptations are poorly understood. Thus, we present a high-quality draft genome of miiuy croaker. The expansions of several gene families which are critical for the fish innate immune system were identified. Compared with the genomes of other fishes, some changes have occurred in the miiuy croaker sensory system including modification of vision and expansion of taste and olfaction receptors. These changes allow miiuy croaker to adapt to the environment during the long-term natural selection. The genome of miiuy croaker may elucidate its relatively well-developed immune defense and provide an adaptation model of the species thriving in turbid deep aquatic environments.

The Sciaenidae, known for their exceptionally large otoliths (sagittal otoliths), are economically important marine fishes and commonly called drum fishes or croakers because of the sounds that these organisms make with their well developed swim bladders^1^. Chinese fishermen create knocking sounds to capture Sciaenidae because of their large otoliths and developed auditory system; as such, a large number of species have been considered as endangered since the 1950 s. Sciaenidae are typically benthic carnivores, and most of these fishes avoid clear waters to live primarily in estuaries, bays, and muddy river banks. However, knowledge about the genetic mechanism of turbid benthic adaptation is limited.

The miiuy croaker *Miichthys miiuy*, one of the representative Sciaenidae, mainly inhabits in the Zhoushan Fisheries located in the estuary of Yangtze River with mud to sandy mud bottoms^2^. In China, this species is an important aquaculture fish that has been widely cultured since the late 1990 s. However, diseases caused by pathogens and parasites have occurred because of high-density feeding; as a consequence, the development of miiuy croaker aquaculture industry has been impeded. The immunity mechanisms of teleosts should be understood to improve fish health. A series of immune-related genes have been identified in this species on the basis of the analyses of transcriptome and EST databases^3^,^4^. To elucidate the immune mechanisms, researchers characterized and comprehensively analyzed several immune-related genes, such as CXC chemokine receptors, toll-like receptors (TLRs) and major histocompatibility complexes (MHCs)^5^,^6^,^7^. As an important link to vertebrate evolution, teleost fish is believed as an important model in the studies on complicated innate immune system and evolutionary origin of the adaptive immune system^8^. With these features, the miiuy croaker is a useful species for understanding the evolution of immune systems and the genetic bases of sensory adaptations of Sciaenidae.

This study presented a high-quality genome sequence and annotation of the miiuy croaker by a whole-genome shotgun approach. Comparative genomic analyses provide insights into the characteristics of the immune system and evolutionary sensory adaptations to the turbid-deep aquatic habitats.

## Results and Discussion

### Genome sequencing and assembly

We performed a whole-genome shotgun strategy to sequence the genome of a wild female miiuy croaker by using an Illumina Hiseq 2000 sequencing platform. We obtained 100.79 Gb high-quality reads from seven pair-end libraries and four mate-pair libraries with various insert sizes ranging from 180 bp to 20 kb after low-quality and duplicated reads were filtered (Table 1, Supplementary Table S1 in Additional file 1). This finding represented an approximately 158-fold coverage of the miiuy croaker genome with an estimated size of 636.22 Mb as indicated by the K-mer frequency method (Supplementary Fig. S1 and Table S2 in Additional file 1). This result is similar to the genome size of 655.26 Mb estimated on the basis of flow cytometry analysis (Supplementary Fig. S2 in Additional file 1). Generated reads were assembled *de novo* by employing the assembler Allpaths-LG^9^, as a result, a draft genome of 619.30 Mb (scaffolds) with contig and scaffold N50 values of 73.32 kb and 1.15 Mb, respectively, was obtained. The largest scaffold measured 20.21 Mb (Supplementary Tables S3 and S4 in Additional file 1).

Soapligner^10^ was applied to realign the high quality short insert size reads onto the assembled scaffolds and to validate the single-base accuracy of the genome assembly by using three different methods. The peak sequencing depth was 127-fold, 92.79% of the genome assembly was more than 50-fold; these results indicated that the genome assembly was highly accurate (Supplementary Fig. S3 in Additional file 1). Aligning the publicly available expressed sequence tags (ESTs)^3^ and transcriptome unigenes^4^ to the assembly with BLAT^11^, we found that the assembly covered 95.81% and 95.59% of the ESTs and unigenes, respectively (Supplementary Table S5 in Additional file 1). The GC content was analyzed to check the randomness of sequencing. The result showed that the miiuy croaker exhibited a pattern similar to that of other fishes; a minor fraction contained less than 20% or more than 80% of the GC content (Supplementary Fig. S4 and Supplementary Table S6 in Additional file 1). Therefore, the similar size and composition of the miiuy croaker to those of other fish genomes and the high coverage level indicated the high quality of our genome assembly.

### Genome characterization and annotation

Good annotation was obtained because of the accurate assembly of the miiuy croaker genome. The *de novo* genome searching^12^ and homology prediction against the RepBase^13^ database revealed that 19.51% of the miiuy croaker genome was comprised of a repeat content, which is similar to that of medaka (17.5%)^14^ and stickleback (25.2%)^15^ (Supplementary Tables S7–S9 in Additional file 1). Of the total repeat contents, 455,319 simple sequence repeats with a total length of 16.28 Mb were identified (Supplementary Table S10 in Additional file 1). With regard to transposable elements (TEs), 51.31 Mb DNA transposons represented the dominant type (8.28% of the genome), followed by long interspersed elements (LINEs, 6.14%) and long terminal repeats (LTRs, 4.45%) (Supplementary Table S8 in Additional file 1). We also identified 1,387,371 single-nucleotide polymorphisms (SNPs) and 382,008 InDels (Supplementary Table S11 in Additional file 1). This result presents a heterozygous rate in the miiuy croaker of 2.24 × 10^−3^, and this rate is higher than that of Atlantic cod (2.09 × 10^−3^)^16^ and stickleback (1.43 × 10^−3^)^15^, but less than that of medaka (3.42 × 10^−2^)^14^, which is the species with the highest rate among the sequenced vertebrates.

After screening out the repetitive contents, we predicted the miiuy croaker genes with *ab inito,* transcriptome-based and homology-based prediction methods. All of the predicted gene structures were integrated with Glean^17^, and a non-redundant gene set containing 21,960 protein-coding genes was generated. The gene set exhibited a higher GC content (52.72%) than that of the whole genome; this finding was also observed in mammals^18^ (Supplementary Tables S12 and S13 in Additional file 1). These genes yielded an average gene and CDS lengths of 12,251.59 bp and 1,789.91 bp, respectively, with an average of 9.82 exons per gene. These genes did not evidently differ from those of other species (Supplementary Fig. S5 and Supplementary Table S14 in Additional file 1). Among these genes, 21,026 (95.75%) were functionally annotated by at least one database (Supplementary Table S15 in Additional file 1); most of these genes revealed significant identities to the sequences in the non-redundant protein (Nr), non-redundant nucleotide (Nt), KOG, and SwissProt databases (Supplementary Fig. S6 in Additional file 1). Furthermore, 93.69% and 80.76% of the protein coding genes with at least one conserved domain could be identified by comparing against InterPro and CDD databases (Supplementary Table S15 in Additional file 1). A total of 15,413 genes were classified into functional categories according to Gene Ontology (GO)^19^ (Supplementary Fig. S7 in Additional file 1) and 11,181 genes were assigned to Kyoto Encyclopedia of Genes and Genomes (KEGG) pathways^20^ (Supplementary Fig. S8 in Additional file 1). In addition, 1,824 non-coding RNA, including 73 rRNA, 522 miRNA, and 1,229 tRNA genes were identified (Supplementary Tables S16 and S17 in Additional file 1). With this complete assembly and well-annotated high quality genome, we can provide a useful resource for the scientific community, comprehensively analyze the genomic features of the miiuy croaker, and comparatively analyze this species with other species.

### Phylogenetic position of the miiuy croaker

**Figure 1:**
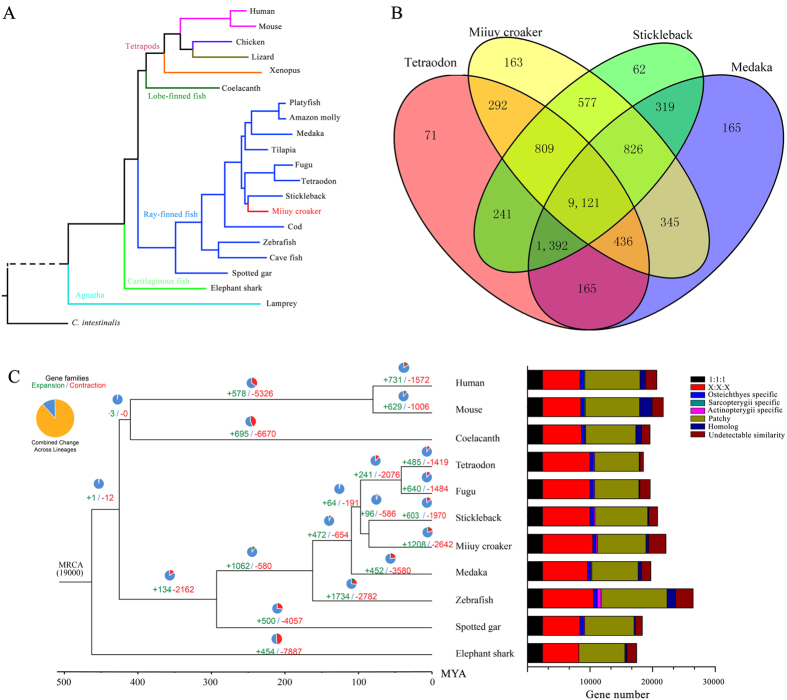
Analysis of the phylogenetic relationship. (**A**) A phylogenetic tree was constructed using 560 single-copy orthologous genes conserved in 21 chordate species and was well supported with high posterior probabilities (PP = 1.00) in all nodes. (**B**) Four species (miiuy croaker, stickleback, medaka and tetraodon) were used to generate the Venn diagram based on the gene family cluster analysis. (**C**) Dynamic evolution and distribution of orthologous gene clusters among 11 vertebrate species. The blue and red numbers represent the expanded and extracted gene families, respectively. MRCA: most recent common ancestor.

In previous studies, the phylogenetic analysis with a mitochondrial genome and multiple nuclear genes suggested that Sciaenidae exhibit a closer affinity to Tetraodontiformes^21^,^22^, however, the phylogenetic relationship based on the genome-scale data set is absent. To investigate the exact phylogenetic position of the miiuy croaker (family Sciaenidae), we compared this species with 20 other chordate species, including 11 teleosts. A phylogenetic tree was constructed using 560 one-to-one orthologs shared with all of the investigated species. The tree showed that stickleback (order Gasterosteiformes) was the closest relative of the miiuy croaker, and the sister group of miiuy croaker and stickleback clades was Tetraodontiformes (Fig. 1A, and Supplementary Fig. S9 in Additional file 1). Furthermore, the estimated time of divergence between miiuy croaker and stickleback was approximately 85.8 million years ago (MYA), which is earlier than the split of tetraodon and fugu from their ancestor (41.8 MYA). The ancestor of miiuy croaker and stickleback split from Tetraodontiformes approximately 97.2 MYA (Supplementary Fig. S9 in Additional file 1).

### Genomic evolution

The gene families in three representative teleost species and in the miiuy croaker genomes were subjected to cluster analysis, and 9,121 gene families were conserved among the four fishes (Fig. 1B). The expansion and contraction analysis of the gene families showed that 1,208 gene families were expanded in the miiuy croaker (Fig. 1C). The significant expansion families (*P* < 0.05) were involved in calcium ion binding (GO:0005509, *P* = 8.20E-27), eye morphogenesis (GO:0048592, *P* = 2.34E-04), and muscle cell development (GO:0055001, *P* = 3.46E-04; Supplementary Table S18 in Additional file 1). These expansions may be associated with the basic life activities, such as calcium metabolism, vision and muscle cell development. The 30 significant contracted gene families contained MHC, which plays a vital role in adaptive immune system^8^, this finding confirmed the partially developed adaptive immunity of the miiuy croaker (Supplementary Table S18 in Additional file 1). The comparison of the gene clusters showed that 5,780 gene families were conserved among all the studied species, including single-copy orthologs (2,392) and many-to-many orthologous (8,034) in the miiuy croaker (Fig. 1C, Supplementary Table S19 in Additional file 1). By comparing the protein sequences of the miiuy croaker with representative Sarcopterygii and Actinopterygii species, we identified 18,810 miiuy croaker genes clustered into 12,587 gene families (Supplementary Fig. S10 in Additional file 1). These results indicated that the gene models of the miiuy croaker were similar to those of the other representative well-annotated vertebrates. We further confirmed that the miiuy croaker species-specific genes and they were enriched with kinase activity (GO:0016301, *P* = 4.30E-07), innate immune response (GO:0045087, *P* = 2.32E-06), and immune effector process (GO:0002252, *P* = 7.08E-04; Supplementary Table S20 in Additional file 1).

### Characterization of the miiuy croaker immune system

**Figure 2:**
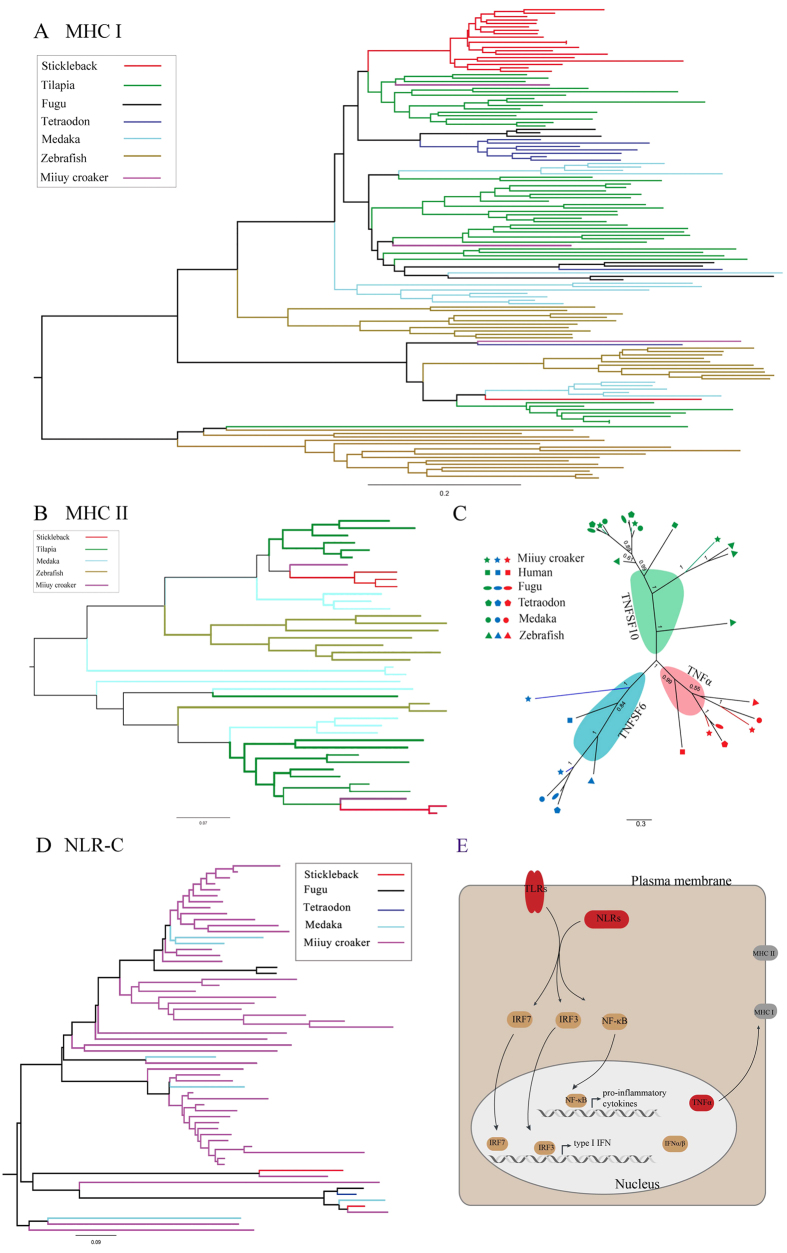
The well-developed innate immunity of the miiuy croaker. (**A**) The phylogenetic tree of the MHC class I protein complex and (**B**) the MHC class II protein complex in different teleosts. (**C**) A phylogenetic tree of the TNF family in the miiuy croaker and five representative vertebrates. (**D**) The phylogenetic tree of the NLR-C family in the miiuy croaker and four teleosts. (**E**) Several key genes are changed in the immunity pathways of the miiuy croaker. The expanded genes, contracted genes and the genes similar with other teleosts are present in red, gray and brown, respectively.

We searched for the immune-related genes in the miiuy croaker genome and only identified two MHC I genes and two MHC II genes, which were much fewer than those in the other sequenced teleosts (Fig. 2A,B). In addition, various interleukins (ILs) belong to γ_c_ cytokine family in fish, including IL-2, IL-4, IL-7, IL-9, IL-15, and IL-21; these ILs play crucial roles in a wide range of adaptive immunity responses^23^. Among these ILs, only IL-15 was identified in the miiuy croaker. The contraction of the MHC gene families and the loss of adaptive immune-related ILs may indicate that the adaptive immunity of the miiuy croaker is not effective. However, the miiuy croaker has evolved a well-developed innate immunity compared with its adaptive immunity. We observed the expansions of tumor necrosis factors (TNFs), which are critical innate cytokines in normal physiology, inflammation response and tumor regression^24^. Since the discovery of TNFs, these cytokines have extensively investigated because of their various functions in the innate immune system. We identified two TNFα, two TNFSF6 and three TNFSF10 in the miiuy croaker, and the number of these TNFs in the miiuy croaker is more than that in other fishes. This result suggested that the miiuy croaker is equipped with exceptional innate immunity in certain domains (Fig. 2C). Furthermore, 50 NOD-like receptor type C (NLR-C) genes were identified in the miiuy croaker. This result indicated the expansion of the NLR family compared with other teleosts except zebrafish (Fig. 2D). NLRs are a recently identified family of cytoplasmic pattern recognition receptors that play an important role in recognizing pathogens in innate immunity^25^. In addition, two TLR2 were discovered whereas one TLR2 was found in other fish. Innate immune genes, such as interferon (IFN) and IFN regulatory factors (IRFs), were also present in the miiuy croaker genome (Fig. 2E). Therefore, the innate immunity of the miiuy croaker is well-developed to compensate for its non-effective adaptive immunity.

### Sensory adaptation to the muddy aquatic environment

To understand adaptation to the muddy habitats, we investigated visual, gustatory, and olfactory-related genes. We found some characteristics that may be beneficial for the survival of the miiuy croaker in muddy environments.

**Figure 3:**
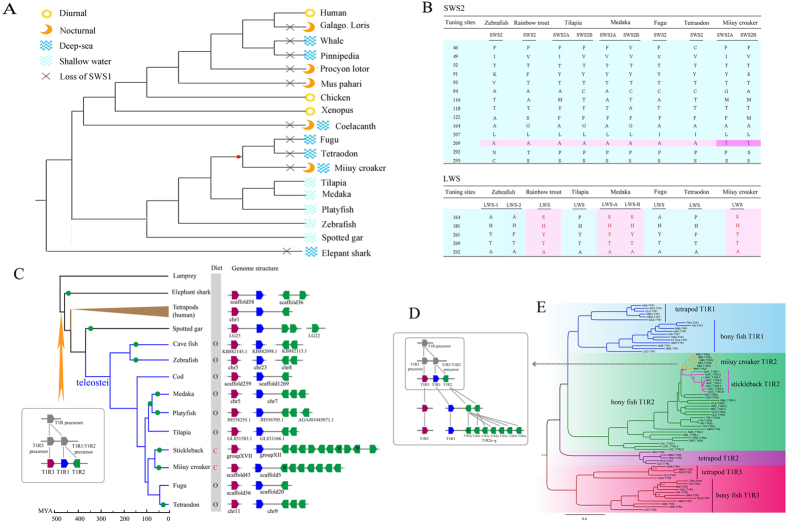
The genetic mechanism of the sensory adaptation to muddy habitats in the miiuy croaker genome. (**A**) The relationship between the natural habitat and the absence of SWS1 in vertebrates. (**B**) Representative amino acid sites involved in the light sensitivity of blue and red opsin compared with seven teleosts. The site numbers are standardized to those of bovine rhodopsin. (**C**) Relationship between T1R2 expansion and dietary habits in teleostei. The green circle represents T1R2 duplication, O represents omnivorous fish, C represents carnivorous fish, and ψ represents pseudogene. (**D**) Hypothesis of T1R2 evolution in the miiuy croaker. According to the phylogenetic analysis we suspect that the T1R1 and T1R2 originated from a common ancestor and three T1R genes from another ancestor. (**E**) Phylogenetic analysis of miiuy croaker T1R2 expansion. We hid the outgroup (zebrafish vomeronasal receptors; V2Rh7 and V2Rx1; the reliability values below 0.85 were noted in figure).

The miiuy croaker is a typical benthic predatory fish living in mud to sandy mud bottoms that seems to have evolved an effective visual system apart from a pair of large eyes. Compared with other fish, certain visual-related genes have been lost, mutated or duplicated in the miiuy croaker. Five classical types of visual pigment genes were identified in fish, including SWS1 (short-wavelength sensitive 1; ultraviolet opsin), SWS2 (short-wavelength sensitive 2; blue opsin), LWS (long-wavelength sensitive; red opsin), RH2 (green opsin), and RH1 (rhodopsin); however only four types were identified in the miiuy croaker, and SWS1 was lost (Supplementary Fig. S12 in Additional file 1). SWS1 is used for ultraviolet vision (UV), so its loss of SWS1 may have resulted from the deep and turbid seawater environment where UV light is not available; turbidity directly limits the transmission of the shortest wavelengths and UV light hardly reaches the deep-sea areas (up to 100 m)^26^. SWS1 might be useless for the miiuy croaker in UV-deficient waters during the natural selection. Coincidentally, deep-sea fish and nocturnal animals with less exposure to UV also lack SWS1 (Fig. 3A). In addition, two RH2 genes (RH2A and RH2B) are generally present in other teleosts, whereas only RH2A is found in the miiuy croaker. Similar to the loss of SWS1, the absence of RH2B was probably because of the weak green light transmission. In addition to the gene loss, gene duplication and mutation occurred in the SWS2 of the amino acid at site 269 (T, threonine); the SWS2 of the miiuy croaker is different from that of other teleosts (A, alanine; Fig. 3B, Supplementary Fig. S13 and Supplementary Tables S21, S22 in Additional file 1). A269T replacement shifted the wavelength of absorption toward the long-wave region of +6 nm (i.e., red shift)^27^,^28^, which may develop a broad field of vision for the miiuy croaker by broadening the spectral breadth of light. Moreover, this shift may be more effective in measuring the distance of the miiuy croaker to its preys or predators in the turbid habitats. Meanwhile, we estimated that the λ_max_ of the LWS gene in the miiuy croaker is about 560 nm, based on five site rules (164, 181, 261, 269, 292)^29^ (Fig. 3B). Additionally, the RH1 underwent duplication but was not limited to the miiuy croaker. Further study found that RH1 may have undergone duplication prior to the divergence of the bony fish from its ancestor of the Neopterygii for better survival in the aquatic environment (Supplementary Fig. S14 in Additional file 1). In summary, three opsin genes, namely, SWS1, RH2, and SWS2 underwent evolutionary changes in the miiuy croaker for more efficient prey capture and defense from predators in the muddy habitats.

Because taste is an important factor for food selection in dietary habits, we searched the miiuy croaker genome for taste receptors and found the expansion of T1R2 gene with seven copies (Fig. 3C). T1R2 and T1R3 form a heterodimer T1R2/T1R3, which functions as a sweet receptor mammals but acts as an umami receptor response to amino acids in fish^30^,^31^. Amino acids are abundant in meat and main umami tastants. Therefore, the expansion T1R2 in the miiuy croaker may be the result of adaptation to its carnivorous diet. The expansion was also observed in stickleback which is an other carnivorous fish^32^. Moreover, we found that carnivorous fish has more T1R2 than omnivorous fish in genome available teleostei (Fig. 3C, Supplementary Table S24 in Additional file 1). To further understand this expansion, subsequent studies on phylogenetics and evolution were performed. According to the result of the phylogenetic analysis results, miiuy croaker T1R2s formed a monophyletic group adjacent to the stickleback T1R2s (Fig. 3E). And gene conversion event was detected in the T1R2s of miiuy croaker and stickleback providing evidence of gene conversion between ancestral sequences of paralogues (Supplementary Table S26 in Additional file 1). This suggested that a successive round of tandem gene duplication before the miiuy croaker diverged from its ancestor and gene conversion after the separation between two species concrtibuted to the expansion. This duplication may have occurred as an adaptation to food selection after the species diverged and the products of the duplication may have originated from two ancestor genes; thus, its probable evolution pattern was deduced (Fig. 3D). Additionally, bony fish T1R2 was grouped with the T1R1 cluster instead of the tetrapod T1R2, which confirms the function difference of tetrapod T1R2 from bony fish T1R2. Besides miiuy croaker and stickleback, many fishes also underwent T1R2 duplication, to better survive in aquatic environments (Fig. 3C).

Olfaction is an indispensable physiological function for detecting food, mates and predators, and is mainly dominated by olfactory receptors (ORs) and vomeronasal receptors (VRs). We identified 113 ORs and 46 VRs in the miiuy croaker, which are more than those of most teleosts (Supplementary Fig. S15 and Supplementary Tables S27, S28 in Additional file 1). It is possible that the abundance of ORs and VRs help miiuy croakers hunt for prey, court for mates, and avoid predators. Taken together, the miiuy croaker has developed a sensory system involving vision, taste, and olfaction, for better survival in the adverse muddy habitats.

## Conclusions

A high-quality genome of a wild miiuy croaker has been successfully assembled and annotated. Our comprehensive and comparative analyses based on the genome sequences of the miiuy croaker enhanced our understanding of the genomic and evolutionary levels of this fish.

A phylogenomic analysis of the miiuy croaker and other species, especially those of sequenced fish genomes such as stickleback, tetraodon and fugu showed that the miiuy croaker is most closely related to stickleback. The contraction of representative adaptive immune genes (MHC) and the expansion of innate immune genes such as TNF and NLR-C were identified. We also found that the genes encoding for other innate immune genes such as TLRs, IFNs, IRFs and ILs were present in the miiuy croaker genome. These results illustrated that the miiuy croaker may have a more developed innate immune system than adaptive immune system, which needs further study.

New insights into the genetic diversity and evolutionary mechanisms may explain the adaptation of the miiuy croaker to its specific aquatic environment. A major sensory adaptation to turbid living conditions includes the loss, mutation of vision-related genes. The loss of SWS1 and RH2B in the miiuy croaker may have resulted from its long-time survival in deep and turbid environments with less exposure to UV and green lights. In addition, the mutation of SWS2 helps the miiuy croaker broaden its field of vision by shifting the wavelength of absorption toward the long-wave region, which may help effectively measure its distance to prey or predators. Moreover, the expansion of T1R2 may have helped the miiuy croaker develop its dietary habits through food selection. Additionally, the miiuy croaker seems to have been equipped with a developed olfactory system with abundant ORs and VRs. Overall, the miiuy croaker has evolved an effective sensory system for better basic activities in muddy habitats.

Our analyses of the miiuy croaker genome provide new insights into its adaptation to the muddy aquatic environment. At the same time, further studies on molecular functions are needed to better understand the survival of the miiuy croaker, which has great significance for aquaculture industries.

## Materials and Methods

### Ethics statement

The study involving live vertebrates was approved by the Ethics Committee of Zhejiang Ocean University. The methods were carried out in accordance with the approved guidelines.

### Sample preparation, sequencing and assembly

A wild female miiuy croaker was caught from the East China Sea area of the Zhejiang Province and was selected for the extraction of DNA from the abdominal muscle for sequencing. Seven pair-end libraries with short insert sizes (180, 300, 600, and 800 bp) and four mate-pair standard libraries with long insert sizes (3, 8, and 20 kb) were constructed according to the Illumina standard protocol. Then the libraries were sequenced using an Illumina Hiseq2000, producing 101 bp or 151 bp reads. Finally, 136.29 Gb of raw data were generated. After filtering, 100.79 Gb of data that had more than 90% of bases with base quality greater than or equal to Q20 remained for the *de novo* assembly. Assembly of the miiuy croaker genome was carried out using the software program Allpaths-LG^9^. The high-quality reads in the seven pair-end libraries with short insert sizes were used to assemble the contigs using the sequence overlap information. By using the distance information of paired-end and mate pair data, SSPACE (version 2.4)^33^ was able to assess the order, distance and orientation of contigs and combine them into scaffolds. Finally, the whole scaffolds were generated after filling the gap (N) regions with Gapcloser^34^. The sequencing depth, GC content distribution and heterozygosity rate of the assembled genome sequence were evaluated by mapping the short insert size reads back to the scaffolds using Soapligner^10^. In addition, ESTs and assembled transcriptome unigenes were mapped to the assembly as reference data for the determination of genomic coverage.

### Gene model prediction and annotation

Tandem repeat sequences in the miiuy croaker genome were identified using the program TRF (Tandem Repeats Finder)^35^. With regard to transposable element (TE) prediction, homology search against the RepBase^13^ TE library using RepeatMasker^36^ and RepeatProteinMask with default parameters was carried out, followed by *de novo* prediction using RepeatModeler. The tRNAs in the genomic sequence were predicted by tRNAscan-SE^37^. BLASTN^38^ was used to identify the rRNAs and miRNAs by aligning the eukarya rRNA sequences from the SILVA^39^ database and miRNA precursor sequences from the miRBase^40^, respectively.

To predict the protein-coding genes in the miiuy croaker genome, homology-based, transcriptome-based and *ab initio* prediction methods were combined. Homology-based prediction was performed by searching against eight related species of proteins using TBLASTN. Then, homologous genome sequences were aligned against the matching proteins using GeneWise to generate gene model structures^41^. Transcriptome reads of the miiuy croaker^4^ were aligned to genomic sequences by Tophat^42^, and transcript structures were obtained using Cufflinks^43^. *Ab initio* prediction was performed by Augustus^44^, GlimmerHMM^45^ and SNAP^46^. The final comprehensive and non-redundant reference gene set was generated by integrating all genes obtained from the *ab initio*, transcriptome-based and homology-based prediction by using Glean^17^.

Annotation of the predicted genes were assigned with the best matched alignment to a number of nucleotide and protein sequence databases, including NT, NR, SwissProt, KOG, and InterPro using BLASTP with an E-value threshold of 1E-5. InterProScan^47^ (Pfam, PRINTS, PROSITE, ProDom, and SMART databases) and NCBI CDD were used to determine the functional motifs and domains in the final gene set. Gene ontology functional classification for these annotated genes was obtained using the annotation retrieved from InterPro. Blast2GO pipeline^48^ was used to describe gene products, and then a web tool WEGO^49^ was used to obtain the GO functional classification of these annotated genes. We also mapped the miiuy croaker protein-coding genes to metabolic pathways and identified the best match for each gene using KAAS based on the KEGG database^50^.

### Gene families and phylogenetic analysis

Miiuy croaker and 20 other chordate proteomes were selected to identify the gene families that descended from a single gene in a common ancestor using OrthoMCL 2.0.9^51^. The longest transcript isoform was selected to represent each gene, and the protein sequences less than 30 amino acids were filtered out, and BLASTP with an e-value cutoff of 1E-5 was used to determine the similarities between genes. Expansion and contraction of gene families was analyzed and processed using CAFE 3.1^52^. The single-copy orthologous genes were aligned by MAFFT 7.205^53^ and concatenated to one super-protein sequence for each species. The concatenated alignment was trimed by Gblocks 0.91b^54^ and the best-fit model (JTT + I + G + F) tested by ProtTest 3.4^55^ was selected. Subsequently, the phylogenetic tree was constructed using RAxML 8.1.5^56^ and MrBayes 3.2^57^. Species divergence time was estimated using the MCMCTREE, implemented in PAML^58^.

### Characteristic analysis of sensory genes

BLASTN and TBLASTN (E-value ≤ 1E-10) were used to search for sensory genes in the miiuy croaker genome. Genes were predicted by GeneWise based on a homology prediction method^41^. Phylogenetic trees were constructed by MrBayes v3.2. Synteny analysis in other species was conducted according to Genomicus^59^ and confirmed by the Ensemble and Map Viewer in NCBI. Detailed methods and analyses are provided in Additional file 1. Gene conversion analysis was performed by GENECONV (version 1.8)^60^ with 10,000 pseudo-replicates and one mismatch allowed (*p* < 0.01).

## Additional Information

**Accession codes:** The whole-genome sequencing project for the miiuy croaker has been deposited in DDBJ/EMBL/GenBank Bioproject database under the accession code JXSJ00000000. The data set of transcript and protein sequences have been deposited in Figshare: ( http://dx.doi.org/10.6084/m9.figshare.2059896).

**How to cite this article**: Xu, T. *et al.* The genome of the miiuy croaker reveals well-developed innate immune and sensory systems. *Sci. Rep.* **6**, 21902; doi: 10.1038/srep21902 (2016).

## Supplementary Material

Supplementary Information

## Figures and Tables

**Table 1:** Statistics of the miiuy croaker genome.

Sequencing	Number	Insert size	Total data (Gb)	Sequence coverage
Paired-end library	7	180–800 bp	76.52	120.27
Mate-pair libraries	3	3 kb–8 kb	11.15	17.53
	1	20 kb	13.12	20.62
Total	11		100.79	158.42
**Assembly**	**Number**	**N50 length**	**Largest length**	**Total length (Mb)**
Contig	21,290	73.32 kb	742.85 kb	594.10
Scaffold	6,294	1.15 Mb	20.21 Mb	619.30
**Annotation**	**Number**	**Total length (Mb)**	**Percentage of genome (%)**	
Repetitive elements	—	120.85	19.51	
Non-coding RNA	1,824	0.17	0.03	
CDS	21,960	39.6	6.35	
