# Supplementary material for: The genome of the miiuy croaker reveals well-developed innate immune and sensory systems
Source: Sci Rep. 2016 Feb 23;6:21902. doi: 10.1038/srep21902 (PMC4763219; doi:10.1038/srep21902)
Supplement: Supplementary Information [file srep21902-s1.doc]

**Supplemental Information**

**The genome of the miiuy croaker reveals well-developed innate immune and sensory systems**

Tianjun Xu^#,*^, Guoliang Xu^#^, Rongbo Che^#^, Rixin Wang^#^, Yanjin Wang, Jinrui Li, Shanchen Wang, Chang Shu, Yuena Sun, Tianxing Liu, Jiang Liu, Aishuai Wang, Jingjing Han, Qing Chu, Qiong Yang

*Laboratory of Fish Biogenetics & Immune Evolution, College of Marine Science,Zhejiang Ocean University, Zhoushan, 316022, China*

# These authors contributed equally to this work.

*Corresponding author. Dr. Tianjun Xu

E-mail: tianjunxu@163.com

**Running title**：Miiuy croaker draft-genome

**Table of content**

[Supplemental Figures 5](#__RefHeading___Toc19846)

[Supplemental Figure S1. The depth distribution of 21-mer in the miiuy croaker genome. 5](#__RefHeading___Toc23692)

[Supplemental Figure S2. Genome size determination of the miiuy croaker by flow cytometry analysis of fluorescently stained nuclei using human erythrocytes as an internal standard. 6](#__RefHeading___Toc24745)

[Supplemental Figure S3. Distribution of sequencing depth of the miiuy croaker assembly. 7](#__RefHeading___Toc4209)

[Supplemental Figure S4. The distributions of GC content in relative fish genomes. 8](#__RefHeading___Toc7595)

[Supplemental Figure S5. Comparison of the gene structure of six sequenced teleost species and mouse genomes. 9](#__RefHeading___Toc28026)

[Supplemental Figure S6. Identity distribution of the hits for miiuy croaker genes against the public databases. 10](#__RefHeading___Toc28683)

[Supplemental Figure S7. Gene Ontology (GO) assignment for the miiuy croaker genes. 11](#__RefHeading___Toc18700)

[Supplemental Figure S8. Kyoto Encyclopedia of Genes and Genomes (KEGG) categories of predicted genes. 12](#__RefHeading___Toc14296)

[Supplemental Figure S9. Phylogenomic and divergence times estimation. 13](#__RefHeading___Toc27197)

[Supplemental Figure S10. Shared gene families of the miiuy croaker with other species. 14](#__RefHeading___Toc21028)

[Supplemental Figure S11. Comparison of protein identity performed between C. intestinalis, four teleosts and two mammals. 15](#__RefHeading___Toc27353)

[Supplemental Figure S12. Comparison of synteny and distribution of four types of visual pigments genes in the miiuy croaker with other vertebrates. 16](#__RefHeading___Toc15805)

[Supplemental Figure S13. Alignment of the amino acid sequences of four types of opsin genes from other teleosts and the miiuy croaker. 17](#__RefHeading___Toc2064)

[Supplemental Figure S14. Vision opsin genes in vertebrates and evolution analysis of RH1 and RH2. 18](#__RefHeading___Toc27919)

[Supplemental Figure S15. Phylogenetic analysis of the olfactory-related gene repertoires. 19](#__RefHeading___Toc29722)

[Supplemental Tables 20](#__RefHeading___Toc30480)

[Supplemental Table S1. Summary of sequencing libraries and data of the miiuy croaker genome. 20](#__RefHeading___Toc14173)

[Supplemental Table S2. The estimation of the miiuy croaker genome size using 21-mer analysis. 21](#__RefHeading___Toc9882)

[Supplemental Table S3. Assembly result of the miiuy croaker genome. 21](#__RefHeading___Toc25962)

[Supplemental Table S4. Summary of genome assembly of the miiuy croaker with other seven sequenced teleost species. 22](#__RefHeading___Toc27793)

[Supplemental Table S5. Evaluating the miiuy croaker assembly using EST data and transcriptome unigenes of the miiuy croaker. 22](#__RefHeading___Toc4297)

[Supplemental Table S6. Statistics of genome content of the miiuy croaker genome. 23](#__RefHeading___Toc10697)

[Supplemental Table S7. Summary of repetitive elements in the miiuy croaker genome. 24](#__RefHeading___Toc30142)

[Supplemental Table S8. Statistics of transposable elements in the miiuy croaker genome. 25](#__RefHeading___Toc5426)

[Supplemental Table S9. Comparison of repeat content from ten sequenced vetebrate speices. 26](#__RefHeading___Toc5197)

[Supplemental Table S10. Length distribution of SSRs based on the number of repeat units. 27](#__RefHeading___Toc2119)

[Supplemental Table S11. Number of SNPs and InDels in the whole genome. 27](#__RefHeading___Toc31591)

[Supplemental Table S12. Gene prediction summary for the miiuy croaker genome. 28](#__RefHeading___Toc3091)

[Supplemental Table S13. Statistics of gene content of the miiuy croaker protein-coding genes. 28](#__RefHeading___Toc22107)

[Supplemental Table S14. Summary of predicted protein-coding genes in the miiuy croaker genome compared with other species. 29](#__RefHeading___Toc4940)

[Supplemental Table S15. The number of genes in the miiuy croaker with homologs or functional assignment from various databases. 29](#__RefHeading___Toc5897)

[Supplemental Table S16. Summary of non-coding RNA distribution and annotation in the miiuy croaker genome. 30](#__RefHeading___Toc29267)

[Supplemental Table S17. Numbers of tRNA isotypes in the whole genome. 31](#__RefHeading___Toc10853)

[Supplemental Table S18. GO terms enriched for expansions and contractions of gene families in the miiuy croaker. 33](#__RefHeading___Toc27668)

[Supplemental Table S19. Comparative analysis of gene clusters among the investigated 11 vertebrates. 34](#__RefHeading___Toc21060)

[Supplemental Table S20. GO enrichment analysis of the gene models specific in the miiuy croaker. 35](#__RefHeading___Toc8998)

[Supplemental Table S21. Representative amino acid sites involved in the light sensitivity of RH1. 36](#__RefHeading___Toc13572)

[Supplemental Table S22. Representative amino acid sites involved in the light sensitivity of RH2. 37](#__RefHeading___Toc4189)

[Supplemental Table S23. GenBank accession numbers of opsin genes in other teleosts used in this study. 38](#__RefHeading___Toc19440)

[Supplemental Table S24. The number T1R and dietary habits in teleostei. 39](#__RefHeading___Toc26014)

[Supplemental Table S25. Accession numbers for the sequences used to phylogenetic analysis. 40](#__RefHeading___Toc30194)

[Supplemental Table S26. Gene conversion analysis for T1R2 gene family of the miiuy croaker and stickleback using coding sequences. 41](#__RefHeading___Toc18125)

[Supplemental Table S27. The number of OR functional genes belonging to different groups in some species. 43](#__RefHeading___Toc16616)

[Supplemental Table S28. Vomeronasal receptor gene repertoire in vertebrates. 43](#__RefHeading___Toc30210)

[Supplemental Notes 44](#__RefHeading___Toc27267)

[1. *De novo* sequencing and assembly of the miiuy croaker genome. 44](#__RefHeading___Toc25181)

[2. Genomic features 45](#__RefHeading___Toc8731)

[3. Comparative genomics and evolution 47](#__RefHeading___Toc6064)

[4. Sensory adaptation to habitats 49](#__RefHeading___Toc22643)

[Supplemental References 50](#__RefHeading___Toc12755)

# Supplemental Figures

**
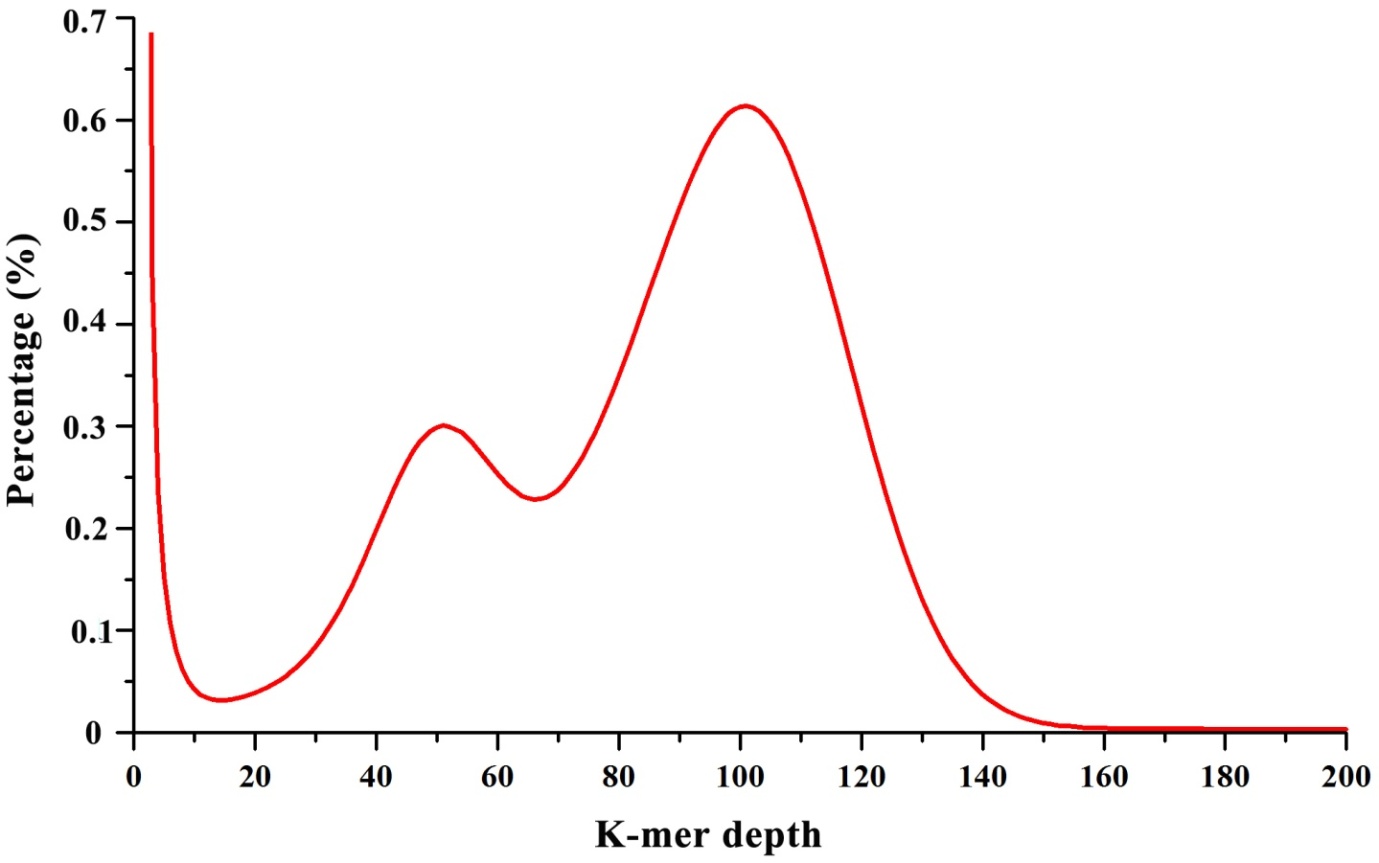
**

Supplemental Figure S1. The depth distribution of 21-mer in the miiuy croaker genome. The X-axis is the depth of K-mers derived from the sequenced reads and Y-axis is the frequency of the K-mer depth. The frequency of each 21-mer was calculated based on the filtered paired-end reads from libraries with short inserts. Two peaks were observed (at 50x and 101x, respectively) indicating the heterozygosity in the miiuy croaker.

**
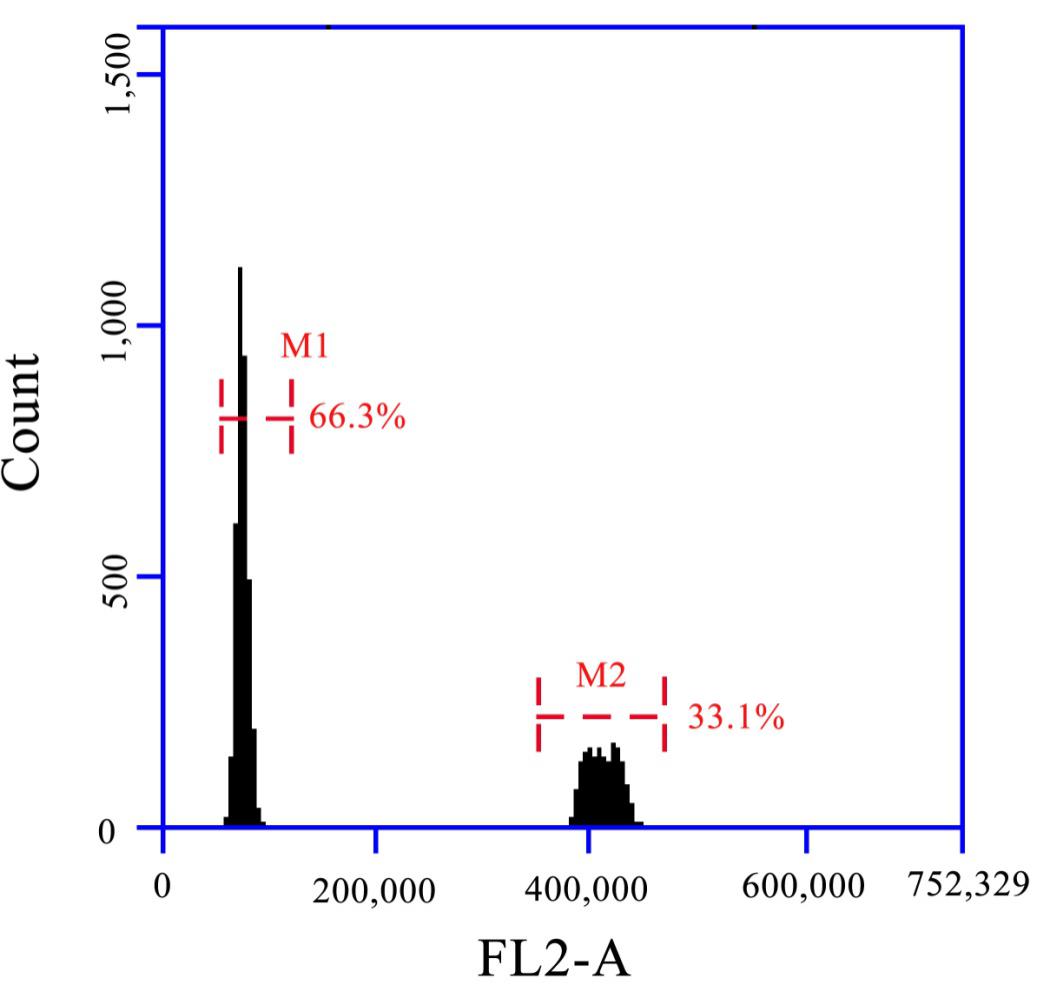
**

Supplemental Figure S2. Genome size determination of the miiuy croaker by flow cytometry analysis of fluorescently stained nuclei using human erythrocytes as an internal standard. The right peak represents the count of human red blood cells and the left peak represents the miiuy croaker sample. X-axis shows the relative fluorescence.

**
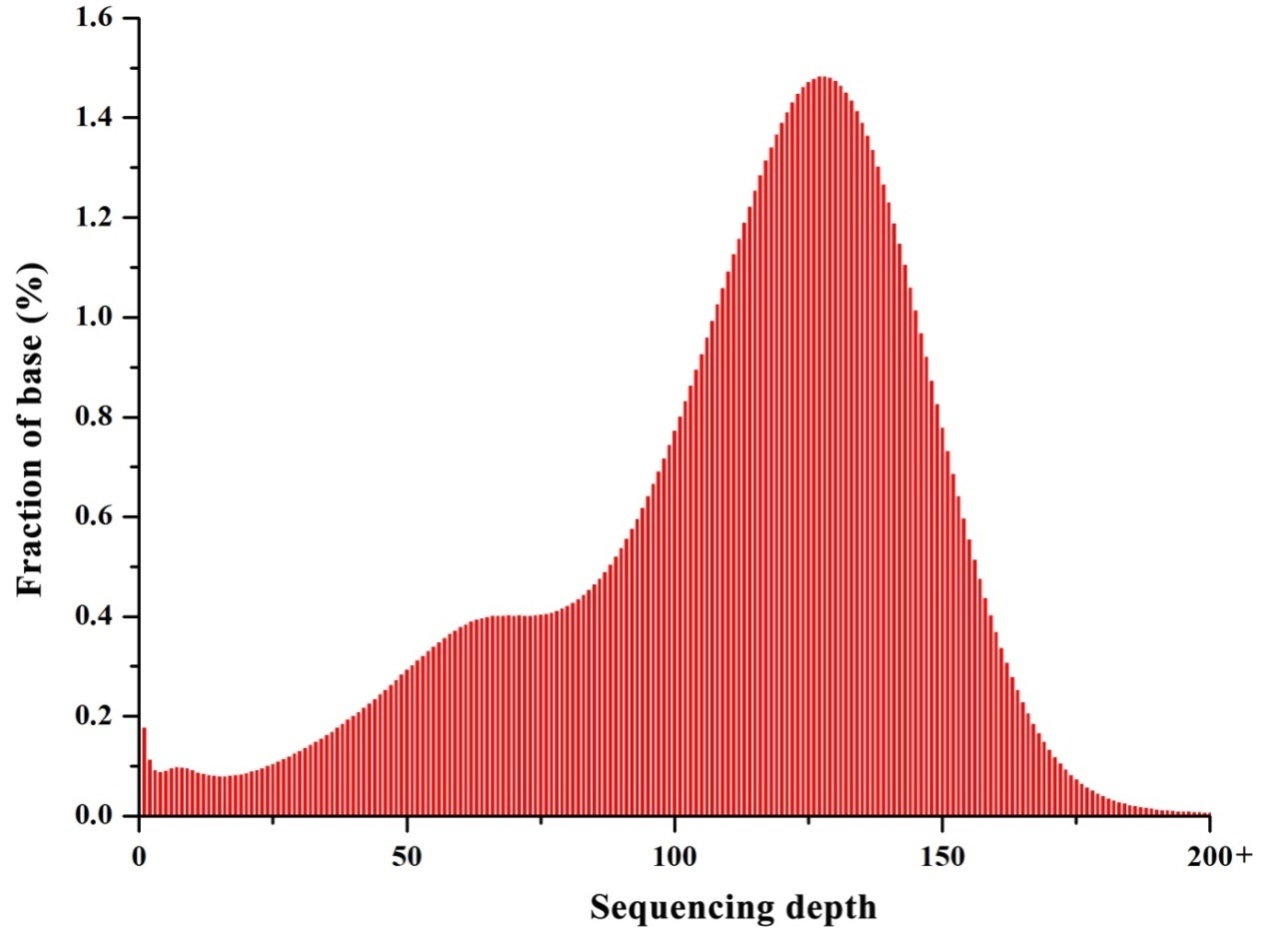
**

Supplemental Figure S3. Distribution of sequencing depth of the miiuy croaker assembly. The depth was obtained by mapping all high quality short insert reads (180 bp and 800 bp) to the assembly and calculating the number of reads across each base.

**
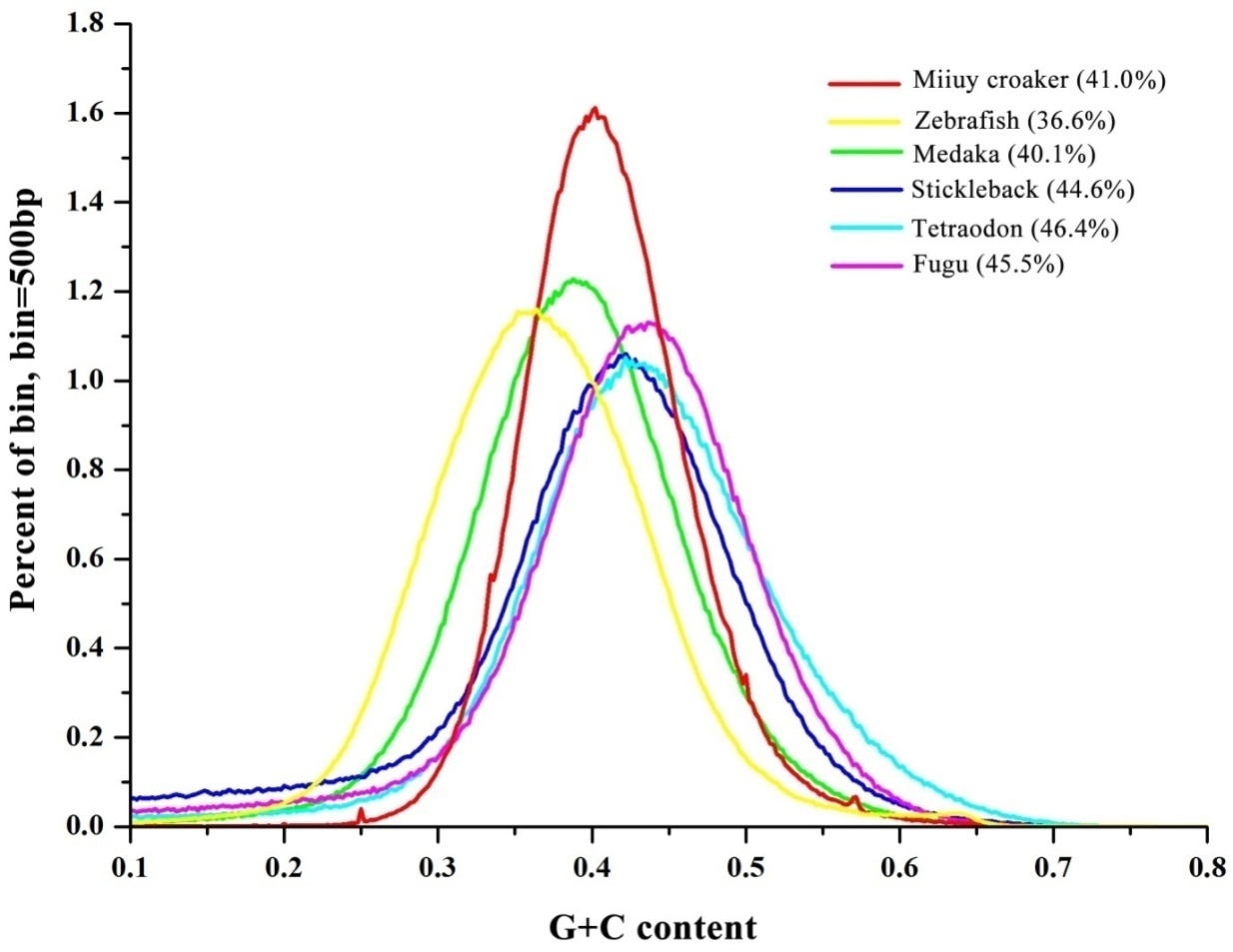
**

Supplemental Figure S4. The distributions of GC content in relative fish genomes. The X-axis is GC content and the Y-axis is the proportion of the bins number divided by the total windows.

**
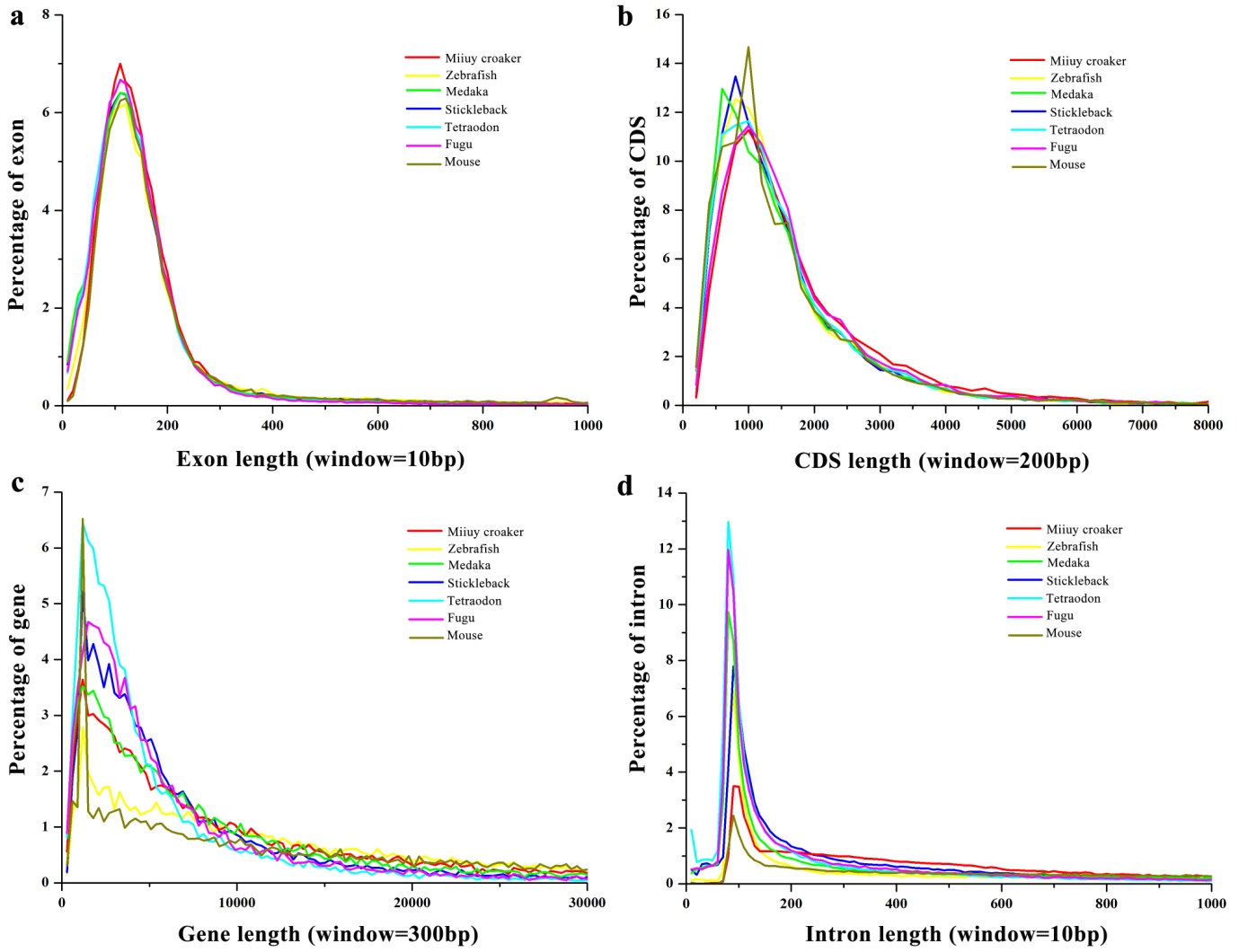
**

Supplemental Figure S5. Comparison of the gene structure of six sequenced teleost species and mouse genomes. **a**) Exon length. **b**) CDS length. **c**) Gene length. **d**) Intron length. No obvious differences were seen for the miiuy croaker compared to the other teleosts, suggesting the high quality annotation of miiuy croaker genes.

**
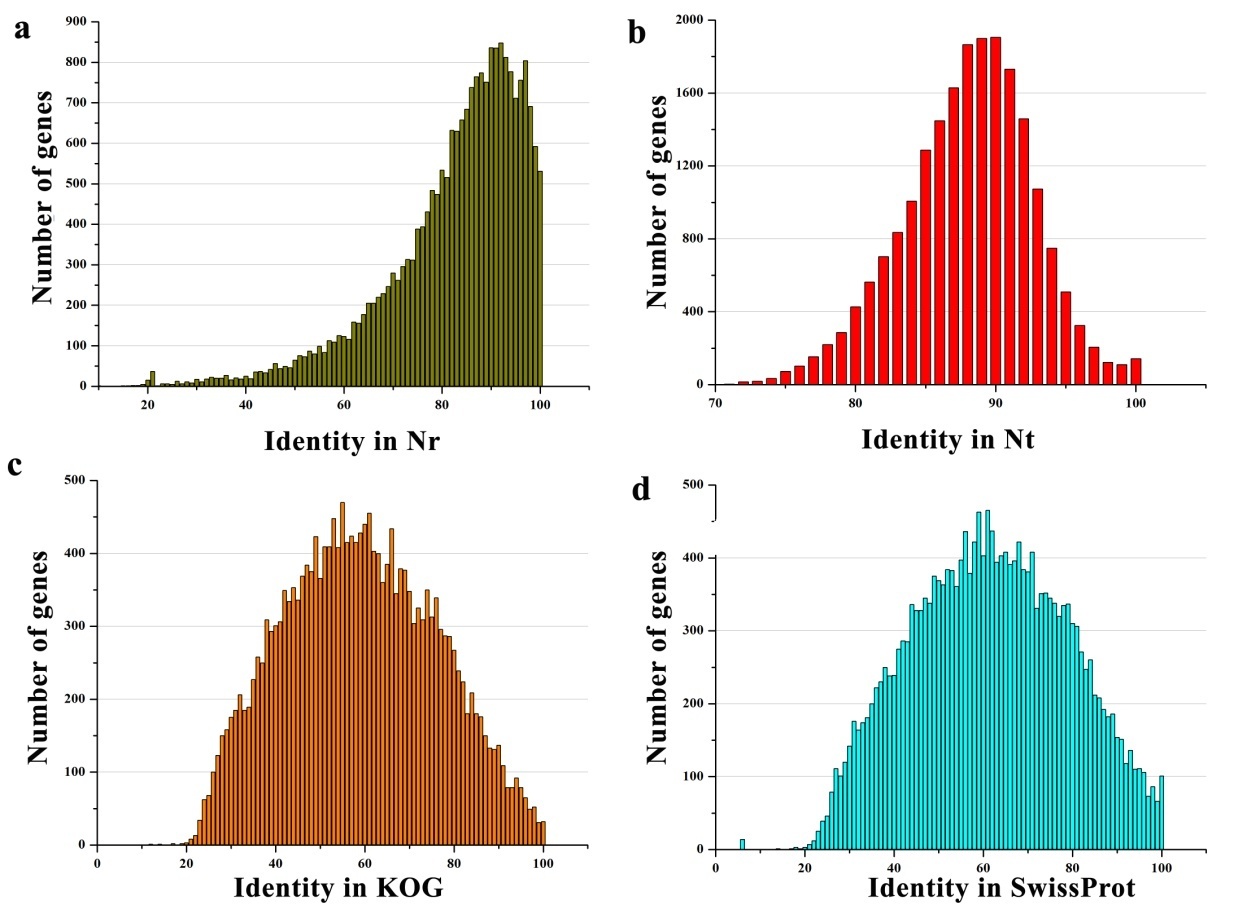
**

Supplemental Figure S6. Identity distribution of the hits for miiuy croaker genes against the public databases. **a**) NR. **b**) NT. **c**) KOG. **d**) SwissProt.

**
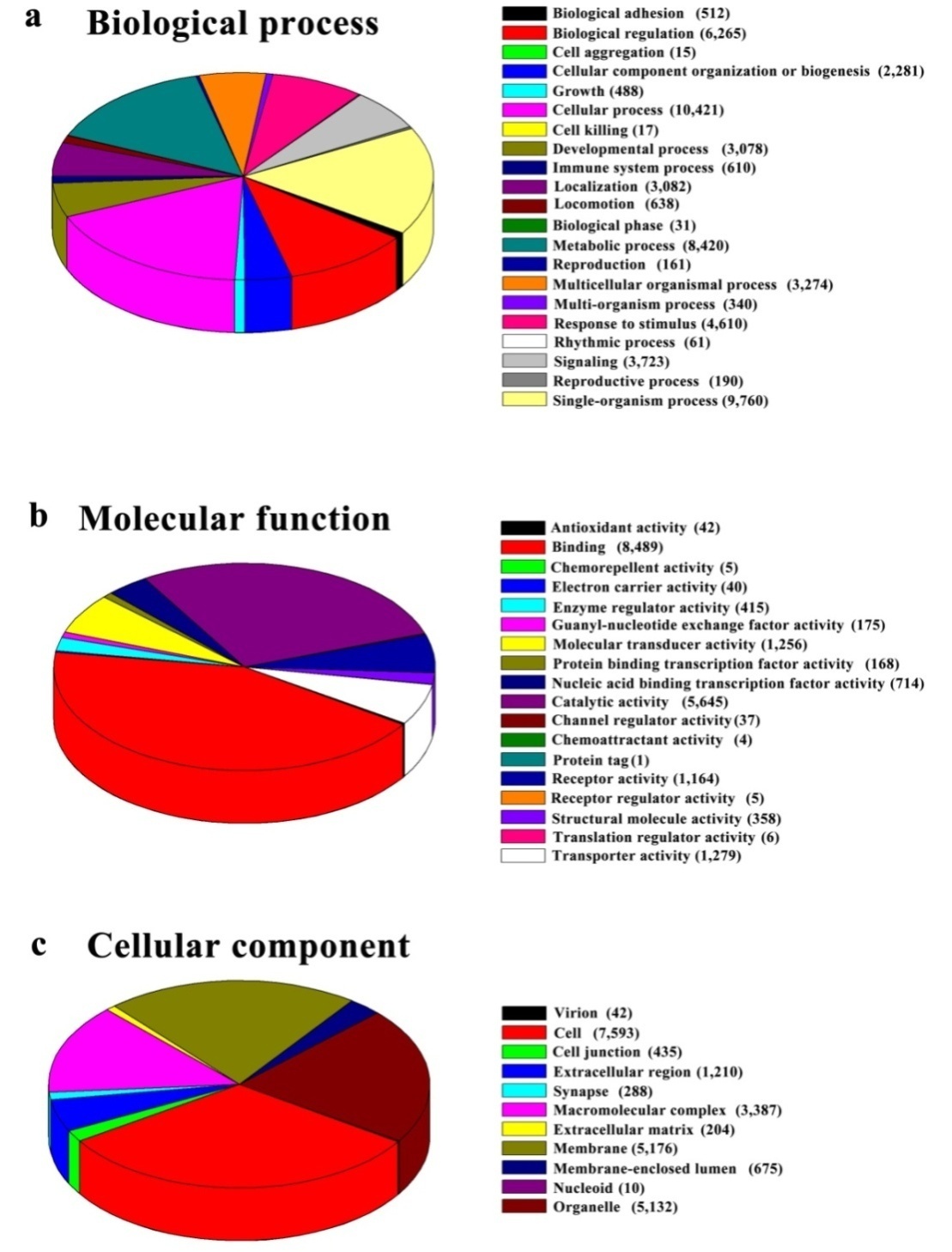
**

Supplemental Figure S7. Gene Ontology (GO) assignment for the miiuy croaker genes. There are 15,576 genes corresponding to at least one GO term, which is represented by the second layer in the GO hierarchy. **a**) Biological processes (BP). **b**) Cellular components (CC). **c**) Molecular functions (MF).

**
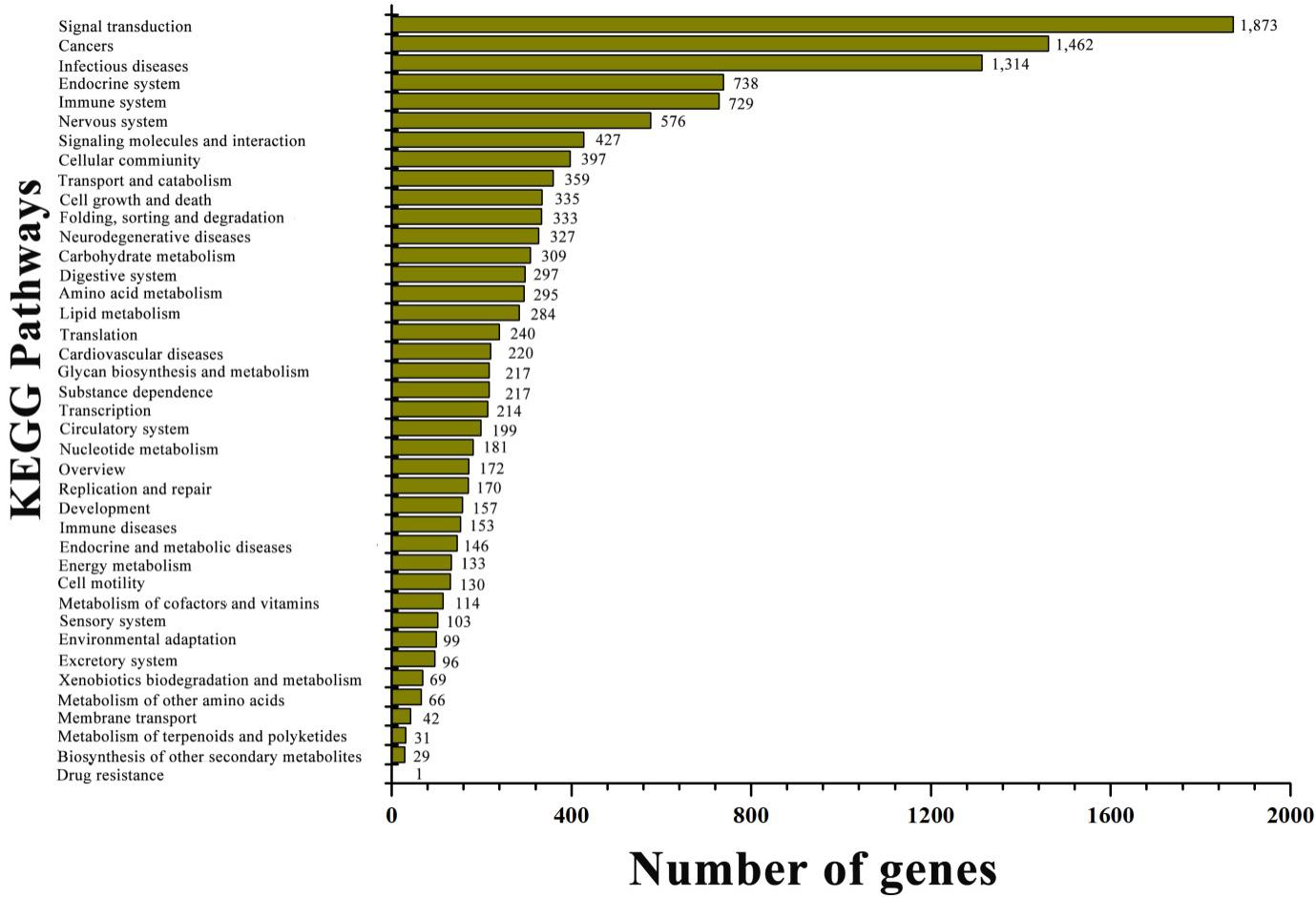
**

Supplemental Figure S8. Kyoto Encyclopedia of Genes and Genomes (KEGG) categories of predicted genes. A total of 11,181 genes were assigned to 342 KEGG pathways summarizing into 40 subgroups.

**
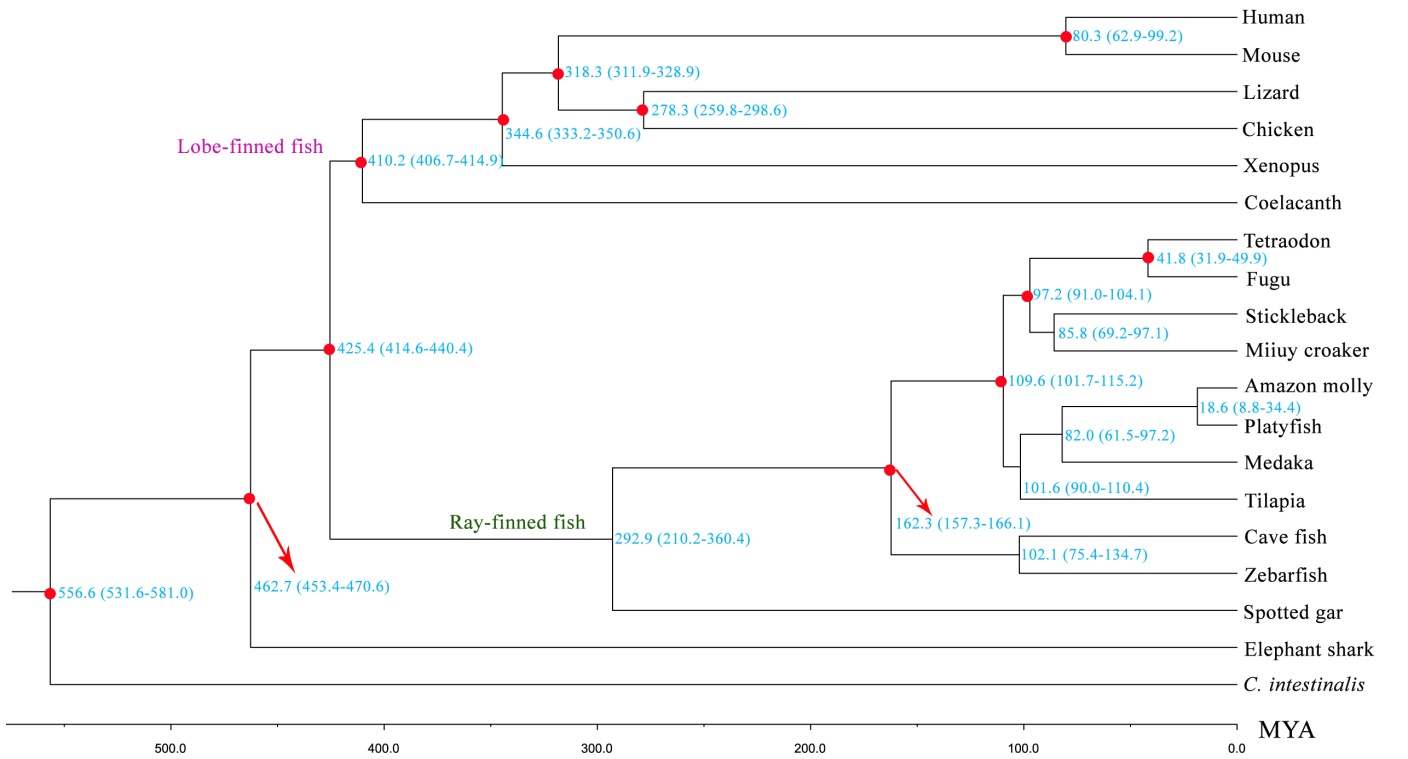
**

Supplemental Figure S9. Phylogenomic and divergence times estimation. The topology was supported by all phylogenetic resources including full-coding sequences, fourfold degenerate sites, and amino acids of920 single-copy genes from 19 vertebrate species. The blue numbers are the estimated divergence time and red dots represent fossil records as calibration times from the TimeTree (http://www.timetree.org/).

**
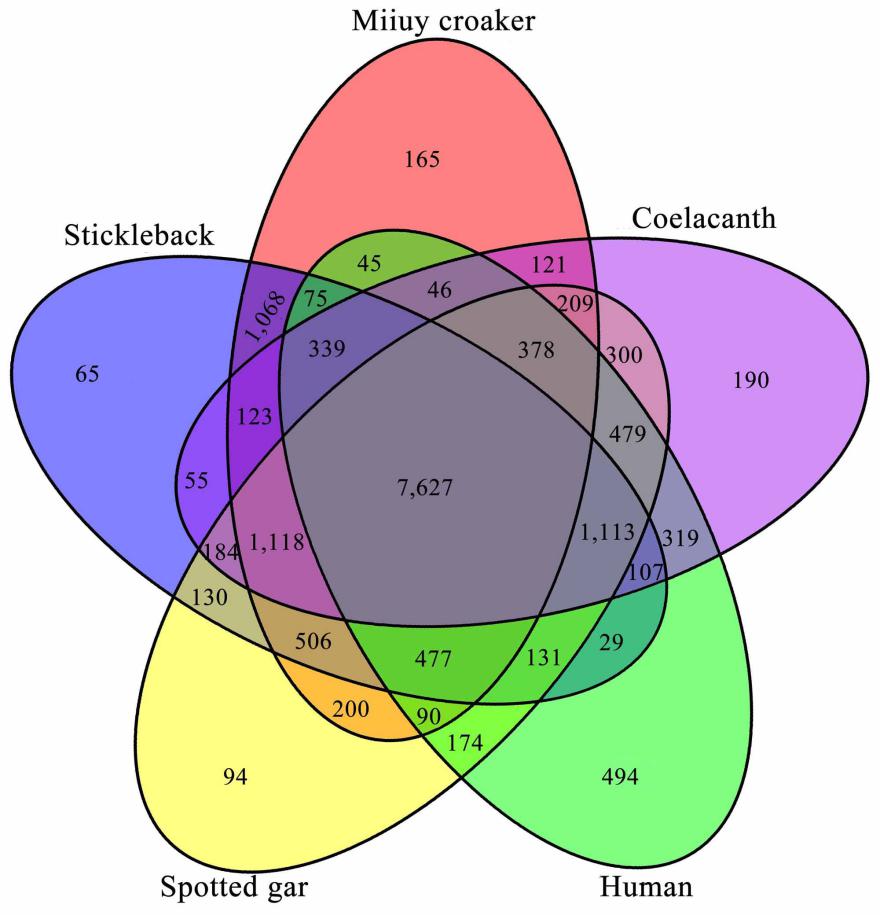
**

Supplemental Figure S10. Shared gene families of the miiuy croaker with other species. Venn diagram of orthologous gene families between miiuy croaker, stickleback, spotted gar, coelacanth and human.

**
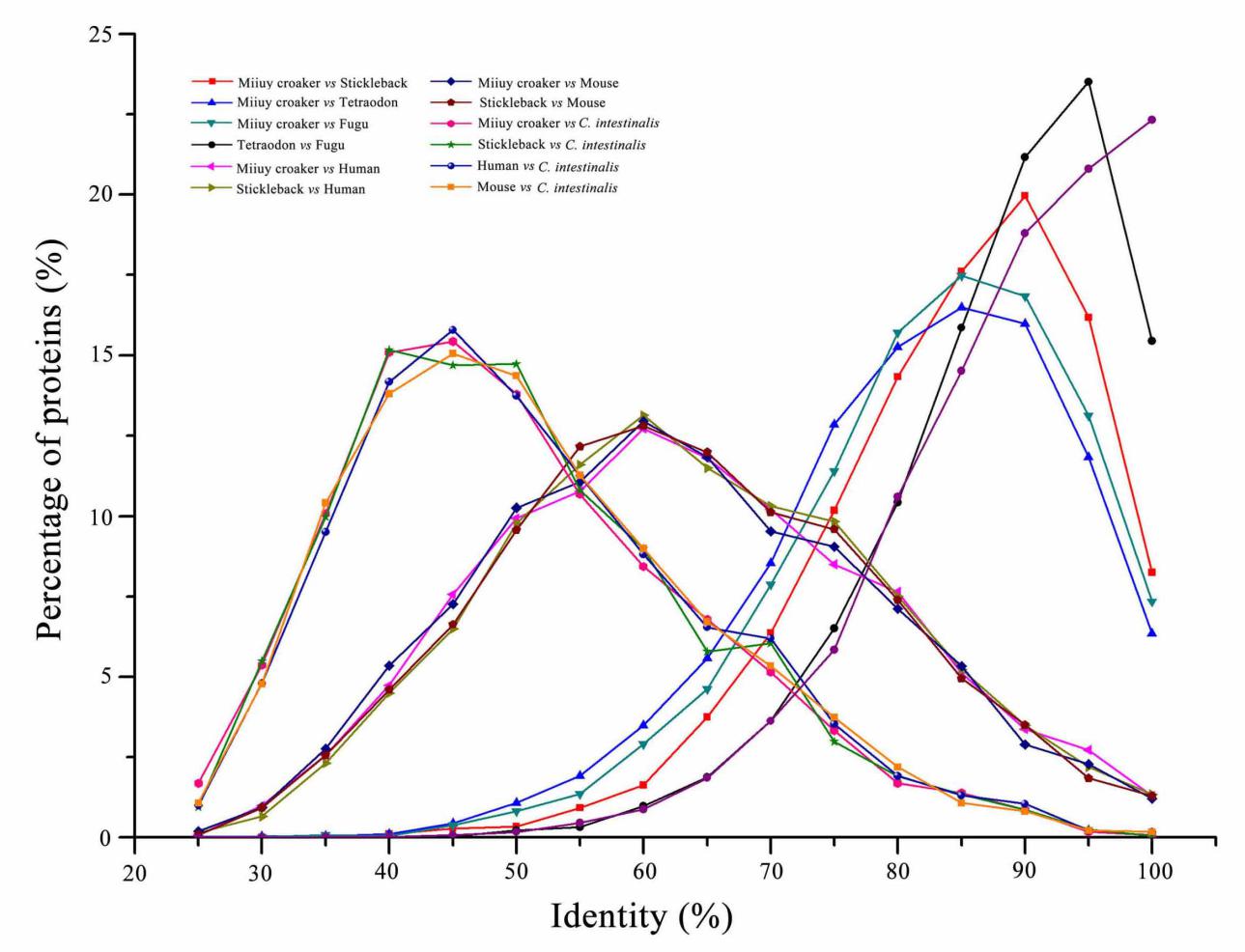
**

Supplemental Figure S11. Comparison of protein identity performed between C. intestinalis, four teleosts and two mammals.

**
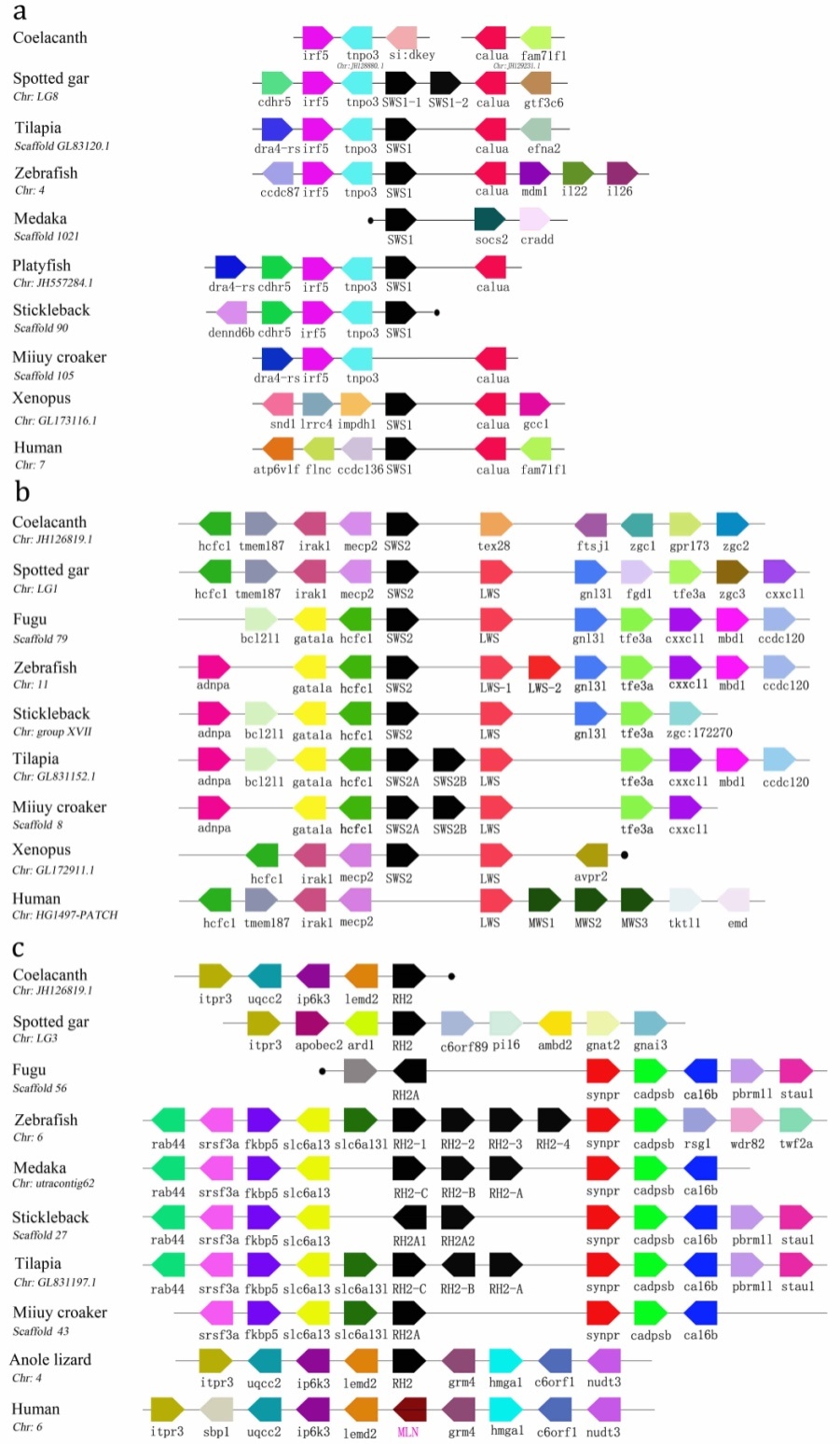
**

Supplemental Figure S12. Comparison of synteny and distribution of four types of visual pigments genes in the miiuy croaker with other vertebrates. **a**) SWS1. **b**) SWS2 and LWS. **c**) RH2. The same color showed the same gene and the grey-colored gene represented the pseudogene. The circles filled in black showed the end of scaffold that the opsin genes located in and the physical distance is ignored.

**
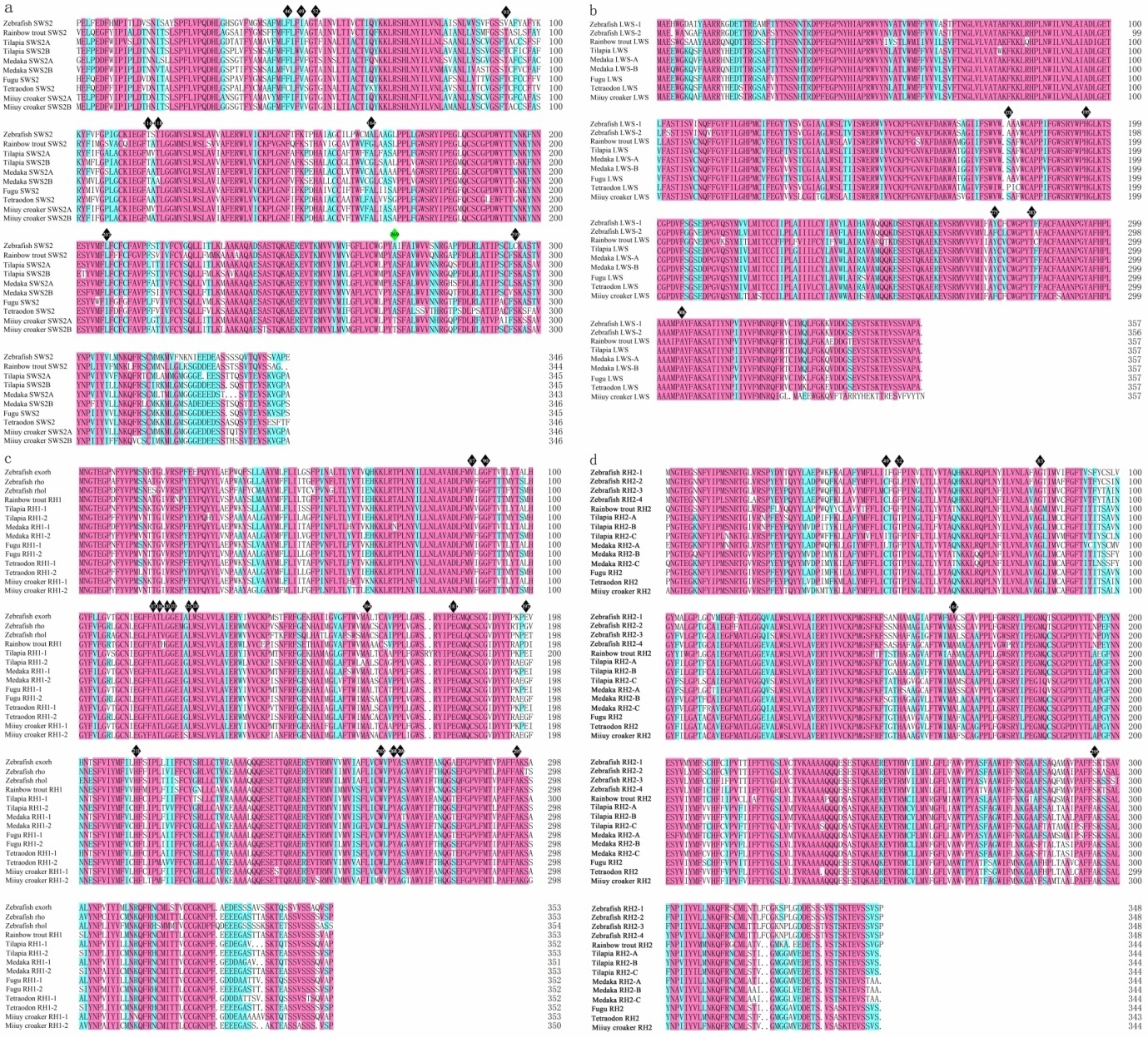
**

Supplemental Figure S13. Alignment of the amino acid sequences of four types of opsin genes from other teleosts and the miiuy croaker. **a**) SWS2. **b**) LWS. **c**) RH1. **d**) RH2. The positions of critical amino acid sites were marked above the relevant sites. The mutated site at 269 was marked in green and black for other sites.

**
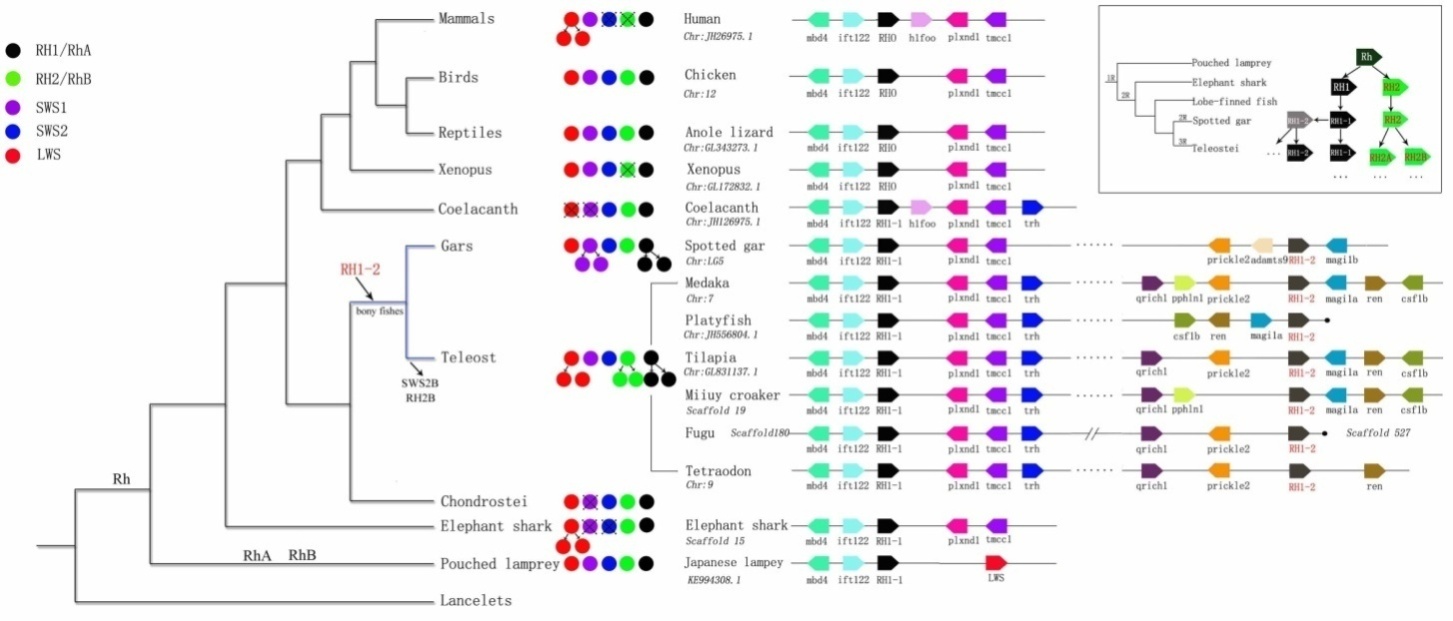
**

Supplemental Figure S14. Vision opsin genes in vertebrates and evolution analysis of RH1 and RH2. The phylogenetic tree showed the duplications and lose of the vision pigment genes. A black cross (×) indicated the gene lose and small arrows (↓or↘) refers to the presence of five types of opsin genes duplications. The apostrophe (…) showed some genes that we ignored and the different scaffold was separated by the symbol (／／).

**
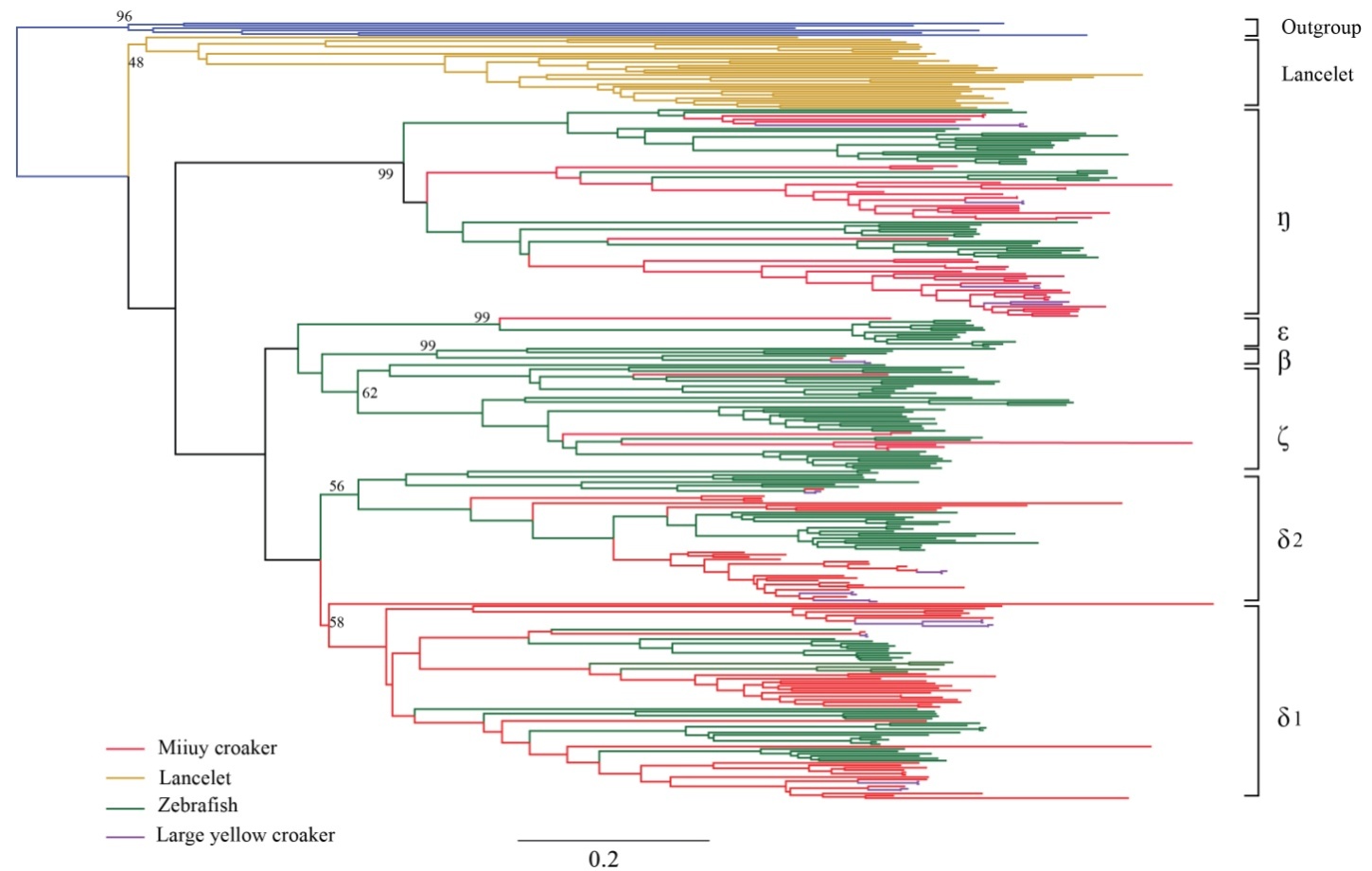
**

Supplemental Figure S15. Phylogenetic analysis of the olfactory-related gene repertoires. The neighbor-joining phylogenetic tree was constructed based on 326 OR-like genes from four fishes and six human non-OR GPCR genes as the outgroup using MEGA 5.0. The Bootstrap values are from 1,000 trials. Different colors represents different species and different OR groups are marked by labels. The bootstrap confidence values are shown at the main nodes of the tree.

# Supplemental Tables

## Supplemental Table S1. Summary of sequencing libraries and data of the miiuy croaker genome.

| **Library** | **Insert size** | **Raw data** | | | | | **High-quality data** | | |
| --- | --- | --- | --- | --- | --- | --- | --- | --- | --- |
|  |  | **Total reads** | **Total bases (bp)** | **GC (%)** | **Q20 (%)** | **Q30 (%)** | **Total reads** | **Total bases (bp)** | **Read length (bp)** |
| DNA-1_1 | 180bp | 135,653,738 | 13,701,027,538 | 42 | 95 | 89 | 109,982,464 | 11,108,228,864 | 101 |
| DNA-1_2-1 | 300bp | 148,057,606 | 14,953,818,206 | 40 | 92 | 84 | 104,202,008 | 10,524,402,808 | 101 |
| DNA-1_2-2 | 300bp | 148,537,382 | 15,002,275,582 | 40 | 92 | 85 | 107,185,260 | 10,825,711,260 | 101 |
| DNA-600 | 600bp | 105,669,734 | 15,956,129,834 | 41 | 94 | 88 | 84,169,360 | 12,709,573,360 | 151 |
| DNA-600_2 | 600bp | 112,042,992 | 16,918,491,792 | 41 | 95 | 89 | 91,062,588 | 13,750,450,788 | 151 |
| DNA_800 | 800bp | 71,132,160 | 10,740,956,160 | 41 | 97 | 89 | 56,600,764 | 8,546,715,364 | 151 |
| DNA-800_2 | 800bp | 76,753,060 | 11,589,712,060 | 41 | 96 | 89 | 59,979,370 | 9,056,884,870 | 151 |
| DNA-1_3 | 3kb | 87,953,904 | 8,883,344,304 | 43 | 92 | 87 | 70,118,820 | 7,082,000,820 | 101 |
| DNA-1_4 | 8kb | 43,464,742 | 4,389,938,942 | 41 | 93 | 86 | 34,136,752 | 3,447,811,952 | 101 |
| DAN-1_5 | 8kb | 7,203,496 | 727,553,096 | 41 | 90 | 85 | 6,122,218 | 618,344,018 | 101 |
| 0713-2 | 20kb | 231,980,748 | 23,430,055,548 | 45 | 79 | 72 | 129,909,556 | 13,120,865,156 | 101 |
| Total |  | 1,168,449,562 | 136,293,303,062 |  |  |  | 853,469,160 | 100,790,989,260 |  |

## Supplemental Table S2. The estimation of the miiuy croaker genome size using 21-mer analysis.

| **K** | **K-mer number** | **K-mer depth** | **Genome Size (bp)** | **Used base (bp)** | **Used read** | **X** |
| --- | --- | --- | --- | --- | --- | --- |
| 21 | 64,258,331,034 | 101 | 636,221,099 | 76,521,967,314 | 613,181,814 | 120 |

## Supplemental Table S3. Assembly result of the miiuy croaker genome.

|  | **Contig** | | **Scaffold** | |
| --- | --- | --- | --- | --- |
|  | **Length (bp)** | **Number** | **Length (bp)** | **Number** |
| **N90** | 13,596 | 9,482 | 82,882 | 1,087 |
| **N80** | 24,846 | 6,282 | 183,612 | 588 |
| **N70** | 37,206 | 4,239 | 323,968 | 330 |
| **N60** | 52,901 | 2,987 | 558,597 | 180 |
| **N50** | 73,323 | 2,035 | 1,145,539 | 95 |
| **Longest** | 742,845 | -- | 20,211,669 | -- |
| **Average** | 27,905 | -- | 98,395 | -- |
| **Total number** | -- | 21,290 | -- | 6,294 |
| **Total size** | 594,102,185 | -- | 619,300,777 | -- |

## Supplemental Table S4. Summary of genome assembly of the miiuy croaker with other seven sequenced teleost species.

| **Species** | **Total length of scaffolds (Mb)** | **Total length of contigs (Mb)** | **Percentage of gaps** | **N50 of scaffolds (bp)** | **N50 of contigs (bp)** | **Number of scaffolds** | **Number of contigs** |
| --- | --- | --- | --- | --- | --- | --- | --- |
| Miiuy croaker | 619 | 594 | 4.06% | 1,145,539 | 73,323 | 6,294 | 21,290 |
| Stickleback | 461 | 447 | 3.23% | 18,115,788 | 83,204 | 1,842 | 16,966 |
| Tetraodon | 358 | 302 | 15.71% | 13,390,619 | 30,260 | 27 | 33,235 |
| Fugu | 393 | 351 | 10.75% | 858,115 | 49,304 | 7,214 | 33,204 |
| Medaka | 869 | 700 | 19.40% | 29,908,082 | 9,628 | 7,189 | 134,399 |
| Cod | 832 | 608 | 26.95% | 136,353 | 2,310 | 398,859 | 555,245 |
| Zebrafish | 1,412 | 1,409 | 0.19% | 54,093,808 | 1,073,451 | 1,133 | 28,972 |

## Supplemental Table S5. Evaluating the miiuy croaker assembly using EST data and transcriptome unigenes of the miiuy croaker.

| **Data type** | **Dataset** | **Number** | **Covered by Assembly (%)** | **With >50% Sequence in one Scaffold** | | **With >90% Sequence in one Scaffold** | |
| --- | --- | --- | --- | --- | --- | --- | --- |
|  |  |  |  | **Number** | **Percent** | **Number** | **Percent** |
| **EST** | All | 3,221 | 95.81% | 2,958 | 91.83% | 2721 | 84.48% |
|  | ≥200bp | 3,134 |  | 2,876 | 91.77% | 2649 | 84.52% |
|  | ≥500bp | 2,371 |  | 2,180 | 91.94% | 2004 | 84.52% |
|  | ≥1000bp | 14 |  | 13 | 92.86% | 13 | 92.86% |
| **Transcripts** | All | 69,071 | 95.59% | 64,010 | 92.67% | 60,439 | 87.50% |
|  | ≥200bp | 59,168 |  | 55,003 | 92.96% | 51,951 | 87.80% |
|  | ≥500bp | 23,634 |  | 22,136 | 93.66% | 20,535 | 86.89% |
|  | ≥1000bp | 9,624 |  | 9,131 | 94.88% | 8,271 | 85.94% |

## Supplemental Table S6. Statistics of genome content of the miiuy croaker genome.

| **Sample ID** | **A** | **T** | **G** | **C** | **N** | **GC** | **Total** |
| --- | --- | --- | --- | --- | --- | --- | --- |
| Number (bp) | 175,431,964 | 175,314,295 | 121,704,398 | 121,655,606 | 25,194,514 | 243,360,004 | 619,300,777 |
| % of genome | 28.32 | 28.30 | 19.65 | 19.64 | 4.06 | 39.30 (40.96 non-N) | 100 |

## Supplemental Table S7. Summary of repetitive elements in the miiuy croaker genome.

| **Type** | **Repeat Size(bp)** | **% of genome** |
| --- | --- | --- |
| **TRF** | 16,279,384 | 2.63 |
| **RepeatMasker** | 29,601,777 | 4.78 |
| **RepeatProteinMask** | 12,187,233 | 1.97 |
| ***De novo*** | 105,539,729 | 17.04 |
| **Total** | 120,847,570 | 19.51 |

## Supplemental Table S8. Statistics of transposable elements in the miiuy croaker genome.

| **Type** | **Repbase TEs** | | **TE Proteins** | | ***De novo*** | | **Combined TEs** | |
| --- | --- | --- | --- | --- | --- | --- | --- | --- |
|  | **Length (bp)** | **%in Genome** | **Length (bp)** | **% in Genome** | **Length (bp)** | **% in Genome** | **Length (bp)** | **% in Genome** |
| **DNA** | 15,604,069 | 2.52 | 2,539,586 | 0.41 | 42,856,697 | 6.92 | 51,305,620 | 8.28 |
| **LINE** | 8,818,869 | 1.42 | 7,494,722 | 1.21 | 32,120,536 | 5.19 | 38,032,210 | 6.14 |
| **LTR** | 5,541,045 | 0.89 | 2,160,886 | 0.35 | 23,439,549 | 3.78 | 27,610,441 | 4.45 |
| **SINE** | 2,386,307 | 0.39 | 0 | 0.00 | 5,892,172 | 0.95 | 7,172,606 | 1.16 |
| **Other** | 12,891 | 0.00 | 0 | 0.00 | 0 | 0.00 | 12,891 | 0.00 |
| **Unknown** | 0 | 0.00 | 0 | 0.00 | 10,370,851 | 1.67 | 10,370,851 | 1.67 |
| **Total** | 29,601,777 | 4.78 | 12,187,233 | 1.97 | 92,526,225 | 14.94 | 101,568,282 | 16.40 |

**Note:** Repbase TEs: the result of RepeatMasker based on Repbase; TE proteins: the result of Repeat ProteinMask based on Repbase; *De novo*: Result of RepeatModeler by using library predicted through *De novo*; Combined TEs: combine the results of Repbase TEs, TE proteins and *De novo*.

## Supplemental Table S9. Comparison of repeat content from ten sequenced vetebrate speices.

| **Species** | **Genome length (Mp)** | **Repeat content from RepeatMasker (%) of genome** | **Repeat content from publication (%) of genome** |
| --- | --- | --- | --- |
| Miiuy croaker | 619 | 4.78 | 19.51 |
| Stickleback | 461 | 7.08 | 25.2 |
| Tetraodon | 359 | 4.6 | -- |
| Fugu | 393 | 7.74 | 11.2 |
| Medaka | 869 | 8.56 | 17.5 |
| Cod | 832 | 10.66 | 25.4 |
| Zebrafish | 1,412 | 49.48 | 52.2 |
| Mouse | 2,717 | 37.69 | 38 |
| Human | 3,096 | 40.08 | 45 |

## Supplemental Table S10. Length distribution of SSRs based on the number of repeat units.

| **Repeat numbers** | **Motif length** | | | | | |  |
| --- | --- | --- | --- | --- | --- | --- | --- |
|  | **Mon** | **Di** | **Tri** | **Tetra** | **Penta** | **Hexa** | **Total** |
| 4 |  |  | 31,566 | 9,797 | 2,335 | 715 | 44,413 |
| 5 |  |  | 11,953 | 3,946 | 958 | 171 | 17,028 |
| 6 |  | 42,496 | 6,269 | 1,921 | 371 | 80 | 51,137 |
| 7 |  | 27,075 | 3,857 | 894 | 268 | 32 | 32,126 |
| 8 |  | 19,848 | 2,874 | 499 | 205 | 9 | 23,435 |
| 9 |  | 15,078 | 1,545 | 350 | 163 | 14 | 17,150 |
| 10 | 54,354 | 12,987 | 955 | 294 | 148 | 2 | 68,740 |
| 11 | 31,836 | 10,901 | 643 | 288 | 127 | 2 | 43,797 |
| 12 | 21,696 | 9,044 | 439 | 254 | 84 | 1 | 31,518 |
| 13 | 15,265 | 7,941 | 357 | 264 | 93 | 1 | 23,921 |
| 14 | 11,906 | 6,336 | 301 | 243 | 76 | 0 | 18,862 |
| 15 | 9,246 | 5,105 | 272 | 254 | 48 | 2 | 14,927 |
| 16 | 7,421 | 4,193 | 238 | 213 | 31 | 0 | 12,096 |
| 17 | 5,600 | 3,590 | 217 | 213 | 40 | 0 | 9,660 |
| 18 | 4,025 | 2,933 | 210 | 206 | 25 | 2 | 7,401 |
| 19 | 2,756 | 2,510 | 142 | 200 | 15 | 0 | 5,623 |
| ≥20 | 8,682 | 20,605 | 1,496 | 2,318 | 355 | 29 | 33,485 |
| Total | 172,787 | 190,642 | 63,334 | 22,154 | 5,342 | 1,060 | 455,319 |

## Supplemental Table S11. Number of SNPs and InDels in the whole genome.

| **Genotype** | **SNP count** | **InDel count** |
| --- | --- | --- |
| Transition |  | 382,008 |
| A-G | 446,525 |
| C-T | 445,102 |
| Transversion |  |
| A-C | 125,723 |
| A-T | 136,531 |
| C-G | 109,159 |
| G-T | 124,331 |
| Total | 1,387,371 |

## Supplemental Table S12. Gene prediction summary for the miiuy croaker genome.

|  | **Gene set** | **Number** | **Average CDS length (bp)** | **Average exon per gene** | **Average exon length (bp)** | **Average intron length (bp)** |
| --- | --- | --- | --- | --- | --- | --- |
| *De novo* | Augustus | 28,744 | 1,427.92 | 7.79 | 183.41 | 1,272.86 |
|  | GlimmerHMM | 96,983 | 785.73 | 5.35 | 147 | 1,122.79 |
|  | SNAP | 60,381 | 1,121.9 | 7.8 | 143.89 | 2,105.86 |
| Homolog | Zebrafish | 28,177 | 1,263.43 | 6.72 | 187.94 | 1,023.55 |
|  | Stickleback | 29,147 | 1,177.22 | 6.69 | 176.08 | 1,003.25 |
|  | Fugu | 24,820 | 1,319.87 | 7.6 | 173.71 | 1,009.86 |
|  | Tetraodon | 23,147 | 1,312.69 | 7.78 | 168.75 | 995.85 |
|  | Medaka | 30,199 | 1,118.31 | 6.27 | 178.26 | 971.32 |
|  | Platyfish | 27,855 | 1,275.66 | 7.13 | 178.91 | 990.02 |
|  | Human | 21,862 | 1,295.48 | 7.47 | 173.4 | 1,049.81 |
|  | Mouse | 21,637 | 1,297.3 | 7.52 | 172.62 | 1,046.05 |
| GLEAN | | 21,960 | 1,789.91 | 9.82 | 181.11 | 1,353.96 |

## Supplemental Table S13. Statistics of gene content of the miiuy croaker protein-coding genes.

| **Sample ID** | **A** | **T** | **G** | **C** | **N** | **GC** | **Total** |
| --- | --- | --- | --- | --- | --- | --- | --- |
| Number (bp) | 10,193,066 | 8,115,899 | 10,479,390 | 10,284,829 | 316,276 | 20,764,219 | 39,389,460 |
| % of CDS | 25.88 | 20.60 | 26.60 | 26.11 | 0.80 | 52.72 | 100 |

Supplemental Table S14. Summary of predicted protein-coding genes in the miiuy croaker genome compared with other species. **(**Ensembl (release version 77)**)**

| **Gene set** | **Genome assembly size (Mb)** | **Number of gene** | **Average CDS length (bp)** | **Average gene length (bp)** | **Average exons number per gene** | **Average exon length (bp)** | **Average intron length(bp)** |
| --- | --- | --- | --- | --- | --- | --- | --- |
| Miiuy croaker | 619 | 21,960 | 1,789.91 | 12,251.59 | 9.82 | 181.11 | 1,353.96 |
| Medaka | 869 | 19,699 | 1,496.02 | 13,080.94 | 10.37 | 154.87 | 1,224.09 |
| Stickleback | 461 | 20,787 | 1,519.25 | 9,183.39 | 10.59 | 161.30 | 779.28 |
| Fugu | 393 | 18,523 | 1,629.03 | 7,810.12 | 10.92 | 151.11 | 606.78 |
| Tetraodon | 358 | 19,602 | 1,510.53 | 6,317.81 | 10.58 | 149.26 | 494.61 |
| Zebrafish | 1,412 | 26,459 | 1,486.73 | 27,625.43 | 9.46 | 239.32 | 2,996.65 |
| Mouse | 2,717 | 21,685 | 1,504.52 | 45,090.92 | 9.14 | 302.59 | 5,094.16 |

## Supplemental Table S15. The number of genes in the miiuy croaker with homologs or functional assignment from various databases.

|  | **Number** | **Percentage (%)** |
| --- | --- | --- |
| Total | 21,960 |  |
| Nt | 20,729 | 94.39 |
| Nr | 20,614 | 93.87 |
| KOG | 20,389 | 92.85 |
| Swissprot | 20,657 | 94.07 |
| InterPro | 20,575 | 93.69 |
| CDD | 17,734 | 80.76 |
| GO | 15,413 | 70.19 |
| KEGG | 11,181 | 50.92 |
| Annotated | 21,026 | 95.75 |
| Unannotated | 934 | 4.25 |

## Supplemental Table S16. Summary of non-coding RNA distribution and annotation in the miiuy croaker genome.

| **Non-coding RNA** | **Type** | **Number** | **Average length (bp)** | **Total length (bp)** | **% of genome** |
| --- | --- | --- | --- | --- | --- |
| rRNA | 8s_rRNA | 69 | 114.45 | 7,897 | 0.001275 |
|  | 18s_rRNA | 2 | 1525 | 3,050 | 0.000492 |
|  | 28s_rRNA | 2 | 2832.5 | 5,665 | 0.000915 |
|  | Total rRNA | 73 | 227.26 | 16,612 | 0.002682 |
| miRNA |  | 522 | 120.02 | 62,648 | 0.001012 |
| tRNA |  | 1,229 | 77.7 | 95,494 | 0.015420 |

## Supplemental Table S17. Numbers of tRNA isotypes in the whole genome.

Numbers of pseudogenes are in parentheses.

| **Amino acid** | **Anticodon** | **Codon** | **tRNA count** |
| --- | --- | --- | --- |
| Ala/A | AGC | GCT | 11 (1) |
|  | CGC | GCG | 20 (3) |
|  | GGC | GCC | 1 |
|  | TGC | GCA | 12 |
| ArgR | ACG | CGT | 9 |
|  | CCG | CGG | 9 (1) |
|  | CCT | AGG | 22 (2) |
|  | TCG | CGA | 15 (2) |
|  | TCT | AGA | 17 |
|  | GCG | CGC | 0 |
| Asn/N | GTT | AAC | 47 (2) |
|  | ATT | AAT | 0 (1) |
| Asp/D | ATC | GAT | 4 (1) |
|  | GTC | GAC | 27 |
| Cys/C | GCA | TGC | 56 |
|  | ACA | TGT | 0 |
| Gln/Q | CTG | CAG | 50 (2) |
|  | TTG | CAA | 23 (6) |
| Glu/E | CTC | GAG | 22 (2) |
|  | TTC | GAA | 29 |
| Gly/G | CCC | GGG | 8 (1) |
|  | GCC | GGC | 18 |
|  | TCC | GGA | 9 |
|  | ACC | GGT | 0 |
| His/H | GTG | CAC | 14 (3) |
|  | ATG | CAT | 0 |
| Ile/I | AAT | ATT | 30 (5) |
|  | GAT | ATC | 3 |
|  | TAT | ATA | 18 (3) |
| Leu/L | AAG | CTT | 20 (1) |
|  | CAA | TTG | 25 (4) |
|  | CAG | CTG | 32 (2) |
|  | TAA | TTA | 13 |
|  | TAG | CTA | 18 |
|  | GAG | CTC | 0 |
| Lys/K | CTT | AAG | 21 |
|  | TTT | AAA | 24 |
| Met/M | CAT | ATG | 140 (78) |
| Phe/F | GAA | TTC | 10 |
|  | AAA | TTT | 0 |
| Pro/P | AGG | CCT | 19 |
|  | CGG | CCG | 7 |
|  | GGG | CCC | 1 |
|  | TGG | CCA | 14 (4) |
| Ser/S | AGA | TCT | 18 |
|  | CGA | TCG | 15 (4) |
|  | GCT | AGC | 19 (5) |
|  | TGA | TCA | 17 (1) |
|  | ACT | AGT | 29 (29) |
|  | GGA | TCC | 1 (1) |
| Thr/T | AGT | ACT | 32 (2) |
|  | CGT | ACG | 16 (5) |
|  | GGT | ACC | 3 (2) |
|  | TGT | ACA | 29 (2) |
| Trp/W | CCA | TGG | 34 (3) |
| Tyr/Y | GTA | TAC | 69 (1) |
| Val/V | AAC | GTT | 9 |
|  | CAC | GTG | 29 (21) |
|  | TAC | GTA | 15 (1) |
|  | GAC | GTC | 0 |
| Stop | TTA | TAA | 1 |
|  | CTA | TAG | 0 |
| SeC | TCA | TGA | 2 |
| SeC(e) | TCA | TGA | 2 |
| SeC(p) | TCA | TGA | 1 |
| Undet | -- | -- | 69 (44) |

## Supplemental Table S18. GO terms enriched for expansions and contractions of gene families in the miiuy croaker.

| **GO-ID** | **Term** | **Category** | | | ***P*-Value** | | | **FDR** | | | |  | |
| --- | --- | --- | --- | --- | --- | --- | --- | --- | --- | --- | --- | --- | --- |
| **Expansions** | |  | | |  | | |  | | |  | | |
| GO:0006334 | nucleosome assembly | | BP | | | 2.23E-64 | | | 1.40E-60 | | | |  |
| GO:0071103 | DNA conformation change | | BP | | | 1.57E-54 | | | 1.64E-51 | | | |  |
| GO:0016459 | myosin complex | | CC | | | 2.04E-31 | | | 1.16E-28 | | | |  |
| GO:0005509 | calcium ion binding | | MF | | | 8.20E-27 | | | 3.43E-24 | | | |  |
| GO:0030286 | dynein complex | | CC | | | 4.40E-19 | | | 1.53E-16 | | | |  |
| GO:0016021 | integral component of membrane | | CC | | | 2.02E-08 | | | 2.85E-06 | | | |  |
| GO:0006200 | ATP catabolic process | | BP | | | 4.12E-06 | | | 3.98E-04 | | | |  |
| GO:0042772 | DNA damage response, signal transduction resulting in transcription | | BP | | | 2.27E-05 | | | 1.80E-03 | | | |  |
| GO:0048593 | camera-type eye morphogenesis | | BP | | | 5.66E-05 | | | 4.07E-03 | | | |  |
| GO:0030511 | positive regulation of transforming growth factor beta receptor signaling pathway | | BP | | | 6.55E-05 | | | 4.59E-03 | | | |  |
| GO:0048592 | eye morphogenesis | | BP | | | 2.18E-04 | | | 1.25E-02 | | | |  |
| GO:0055001 | muscle cell development | | BP | | | 3.46E-04 | | | 1.90E-02 | | | |  |
| GO:0008307 | structural constituent of muscle | | MF | | | 4.71E-04 | | | 2.44E-02 | | | |  |
| GO:0007216 | G-protein coupled glutamate receptor signaling pathway | | BP | | | 8.18E-04 | | | 3.72E-02 | | | |  |
| **Contractions** |  |  | | |  | | |  | | |  | | |
| GO:0006482 | protein demethylation | | | BP | | | 3.24E-15 | | | 1.94E-12 | | | |
| GO:0043044 | ATP-dependent chromatin remodeling | | | BP | | | 3.24E-15 | | | 1.94E-12 | | | |
| GO:0070988 | demethylation | | | BP | | | 2.48E-14 | | | 1.15E-11 | | | |
| GO:0032200 | telomere organization | | | BP | | | 4.84E-14 | | | 2.03E-11 | | | |
| GO:0022403 | cell cycle phase | | | BP | | | 1.56E-13 | | | 6.13E-11 | | | |
| GO:0002244 | hematopoietic progenitor cell differentiation | | | BP | | | 1.12E-11 | | | 3.6E-09 | | | |
| GO:0046982 | protein heterodimerization activity | | | MF | | | 5.94E-11 | | | 1.52E-08 | | | |
| GO:0016570 | histone modification | | | BP | | | 3.68E-08 | | | 7.58E-06 | | | |
| GO:0045596 | negative regulation of cell differentiation | | | BP | | | 7.71E-08 | | | 0.0000148 | | | |
| GO:0046983 | protein dimerization activity | | | MF | | | 1.8E-07 | | | 0.0000318 | | | |
| GO:0048534 | hematopoietic or lymphoid organ development | | | MP | | | 2.83E-06 | | | 0.000439 | | | |
| GO:0042611 | MHC protein complex | | | CC | | | 8.44E-06 | | | 0.00121 | | | |

## Supplemental Table S19. Comparative analysis of gene clusters among the investigated 11 vertebrates.

| **Species** | **1:1:1** | **X:X:X** | **Osteichthyes specific** | **Sarcopterygii specific** | **Actinopterygii specific** | **Patchy** | **Homolog** | **Undetectable similarity** |
| --- | --- | --- | --- | --- | --- | --- | --- | --- |
| Human | 2,392 | 6,014 | 549 | 190 | 0 | 8,833 | 852 | 1,839 |
| Mouse | 2,392 | 6,141 | 517 | 174 | 0 | 8,694 | 1,995 | 1,772 |
| Coelacanth | 2,392 | 6,239 | 496 | 151 | 0 | 8,022 | 913 | 1,356 |
| Fugu | 2,392 | 7,606 | 524 | 0 | 189 | 7,138 | 117 | 557 |
| Tetraodon | 2,392 | 7,606 | 525 | 0 | 182 | 7,105 | 141 | 1,651 |
| Stickleback | 2,392 | 7,595 | 550 | 0 | 221 | 8,452 | 174 | 1,403 |
| Miiuy croaker | 2,392 | 8,034 | 519 | 0 | 213 | 7,776 | 414 | 2,612 |
| Medaka | 2,392 | 7,186 | 517 | 0 | 185 | 7,395 | 525 | 1,499 |
| Zebrafish | 2,392 | 8,172 | 585 | 0 | 620 | 10,536 | 1,357 | 2,797 |
| Spotted gar | 2,392 | 6,007 | 537 | 0 | 173 | 7,921 | 192 | 1,119 |
| Elephant shark | 2,392 | 5,812 | 0 | 0 | 0 | 7,349 | 337 | 1,560 |

“1:1:1” represents shared single-copy genes. “X:X:X” indicates any other conserved orthologous groups in all studied species.“Patchy”indicates other orthologs that are present in at least one Actinopterygii and one Sarcopterygii genome. “Homology” indicates partial homology detected with E < 10-5 but no orthology classified.

## Supplemental Table S20. GO enrichment analysis of the gene models specific in the miiuy croaker.

| **GO-ID** | **Term** | **Category** | ***P*-Value** | **FDR** |
| --- | --- | --- | --- | --- |
| GO:0043234 | protein complex | CC | 5.25E-10 | 2.06E-07 |
| GO:0002429 | immune response-activating cell surface receptor signaling pathway | BP | 1.77E-08 | 4.81E-06 |
| GO:0050778 | positive regulation of immune response | BP | 3.11E-08 | 8.14E-06 |
| GO:0070062 | extracellular vesicular exosome | CC | 1.91E-07 | 4.12E-05 |
| GO:0016301 | kinase activity | MF | 4.30E-07 | 7.71E-05 |
| GO:0097193 | intrinsic apoptotic signaling pathway | BP | 4.88E-07 | 8.27E-05 |
| GO:0031098 | stress-activated protein kinase signaling cascade | BP | 1.62E-06 | 2.18E-04 |
| GO:0007204 | positive regulation of cytosolic calcium ion concentration | BP | 1.63E-06 | 2.18E-04 |
| GO:0045087 | innate immune response | BP | 2.32E-06 | 2.91E-04 |
| GO:0001666 | response to hypoxia | BP | 3.28E-06 | 3.71E-04 |
| GO:0022838 | substrate-specific channel activity | BP | 5.42E-06 | 5.53E-04 |
| GO:0038093 | Fc receptor signaling pathway | BP | 5.61E-06 | 5.63E-04 |
| GO:0010309 | acireductone dioxygenase [iron(II)-requiring] activity | MF | 1.83E-05 | 1.47E-03 |
| GO:0051283 | negative regulation of sequestering of calcium ion | BP | 2.52E-05 | 1.83E-03 |
| GO:0002433 | immune response-regulating cell surface receptor signaling pathway involved in phagocytosis | BP | 2.56E-05 | 1.83E-03 |
| GO:0014808 | release of sequestered calcium ion into cytosol by sarcoplasmic reticulum | BP | 3.47E-05 | 2.30E-03 |
| GO:0051251 | positive regulation of lymphocyte activation | BP | 6.06E-05 | 3.52E-03 |
| GO:0002696 | positive regulation of leukocyte activation | BP | 6.06E-05 | 3.52E-03 |
| GO:0007399 | nervous system development | BP | 8.56E-05 | 4.49E-03 |
| GO:0034704 | calcium channel complex | CC | 1.16E-04 | 5.62E-03 |
| GO:0060316 | positive regulation of ryanodine-sensitive calcium-release channel activity | BP | 3.09E-04 | 1.18E-02 |
| GO:0002252 | immune effector process | BP | 7.08E-04 | 2.26E-02 |
| GO:0005513 | detection of calcium ion | BP | 1.48E-03 | 3.73E-02 |

## Supplemental Table S21. Representative amino acid sites involved in the light sensitivity of RH1.

| **Tuning sites** | **Zebrafish** | | | **Rainbow trout** | **Tilapia** | | **Medaka** | | **Fugu** | | **Tetraodon** | | **Miiuy croaker** | |
| --- | --- | --- | --- | --- | --- | --- | --- | --- | --- | --- | --- | --- | --- | --- |
|  | exorh | rho | rhol | RH1 | RH1-1 | RH1-2 | RH1-1 | RH1-2 | RH1-1 | RH1-2 | RH1-1 | RH1-2 | RH1-1 | RH1-2 |
| 87 | V | V | V | V | V | V | I | V | V | V | V | V | I | V |
| 90 | G | G | G | G | G | G | G | G | G | G | G | G | G | G |
| 117 | A | A | A | A | A | A | A | A | A | A | A | A | A | A |
| 118 | T | T | T | T | T | T | T | T | T | T | T | T | T | T |
| 120 | G | G | G | G | G | G | G | G | G | G | G | G | G | G |
| 121 | G | G | G | G | G | G | G | G | G | G | G | G | G | G |
| 125 | L | L | L | L | L | L | L | L | L | L | L | L | L | L |
| 126 | W | W | W | W | W | W | W | W | W | W | W | W | W | W |
| 164 | A | A | A | A | A | A | A | A | A | A | A | A | A | A |
| 181 | E | E | E | E | E | E | E | E | E | E | E | E | E | E |
| 197 | E | G | E | D | E | G | E | G | E | G | E | G | E | G |
| 211 | H | H | H | H | H | H | H | H | H | H | H | H | H | H |
| 265 | W | W | W | W | W | W | W | W | W | W | W | W | W | W |
| 268 | Y | Y | Y | Y | Y | Y | Y | Y | Y | Y | Y | Y | Y | Y |
| 269 | A | A | A | A | A | A | A | A | A | A | A | A | A | A |
| 295 | A | A | A | A | A | A | A | A | A | A | A | A | A | A |

## Supplemental Table S22. Representative amino acid sites involved in the light sensitivity of RH2.

| **Tuning sites** | **Zebrafish** | | | | **Rainbow trout** | **Tilapia** | | | **Medaka** | | | **Fugu** | **Tetraodon** | **Miiuy croaker** |
| --- | --- | --- | --- | --- | --- | --- | --- | --- | --- | --- | --- | --- | --- | --- |
|  | RH2-1 | RH2-2 | RH2-3 | RH2-4 | RH2 | RH2-A | RH2-B | RH2-C | RH2-A | RH2-B | RH2-C | RH2 | RH2 | RH2 |
| 49 | I | C | C | C | C | C | C | I | L | C | C | C | C | C |
| 52 | F | L | F | F | F | T | T | F | F | T | T | T | T | T |
| 83 | G | G | G | G | G | G | G | G | G | G | G | G | G | G |
| 164 | A | A | A | A | A | A | A | A | A | A | A | A | A | A |
| 295 | S | S | S | S | S | A | A | S | S | A | A | A | A | A |

## Supplemental Table S23. GenBank accession numbers of opsin genes in other teleosts used in this study.

| **Gene** | **Species** | **Accession no.** |
| --- | --- | --- |
| RH1 | Zebrafish | AB087811, JQ614147, NM_131084 |
|  | Rainbow trout | NM_001124319 |
|  | Tilapia | XM_003439005, ENSORLG00000010979 |
|  | Medaka | AB180742, NM_001104695 |
|  | Fugu | AF137214, NM_001078631 |
|  | Tetraodon | CAAE01015033, ENSTNIG00000017925 |
| RH2 | Zebrafish | NM_131253, NM_182891, NM_182892, NM_131254 |
|  | Rainbow trout | NM_001124323 |
|  | Tilapia | JF262086 |
|  | Medaka | AB223053, AB223054, AB223055 |
|  | Fugu | NM_001033712 |
|  | Tetraodon | AY598944 |
| SWS2 | Zebrafish | NM_131192 |
|  | Rainbow trout | NM_001124322 |
|  | Tilapia | JF262088 |
|  | Medaka | AB223056, AB223057 |
|  | Fugu | AY598947 |
|  | Tetraodon | CAAE01014528 |
| LWS | Zebrafish | NM_001002443.1, NM_131175.1 |
|  | Rainbow trout | NM_001124320.1 |
|  | Tilapia | AF247128.1 |
|  | Medaka | AB223052.1, AB223051.1 |
|  | Fugu | AY598942.1 |
|  | Tetraodon | AY598943.1 |

## Supplemental Table S24. The number T1R and dietary habits in teleostei.

| **Species** | **T1R number** | | | | **Dietary habits^a^** | |  |
| --- | --- | --- | --- | --- | --- | --- | --- |
|  | **T1R1** | **T1R2(pseudogenes)** | | **T1R3** | |  | |
| Cave fish | 1 | 2 | 1 | | O | |  |
| Zebrafish | 1 | 2 | 1 | | O | |  |
| Cod | 1 | 1 | 1 | | O | |  |
| Fugu | 1 | 1 | 1 | | O | |  |
| Tetraodon | 1 | 3 | 1 | | O | |  |
| Platyfish | 1 | 3 | 1 | | O | |  |
| Medaka | 1 | 3 | 1 | | O | |  |
| Tilapia | 1 | 2 | 2 | | O | |  |
| Stickleback | 1 | 8(2) | 1 | | C | |  |
| Miiuy croaker | 1 | 6(1) | 1 | | C | |  |

a: Data are from fishbase ([http://www.fishbase.org](http://www.fishbase.org/search.php))and Animal Diversity Web (<http://animaldiversity.ummz.umich.edu/>).

## Supplemental Table S25. Accession numbers for the sequences used to phylogenetic analysis.

| **Genes*** | **Accession numbers** | **Genes** | **Accession numbers** |
| --- | --- | --- | --- |
| DRE-V2RX1 | NM_001082899.1 | XMA_T1R2-3 | ENSXMAT00000018700 |
| DRE-V2RH7 | NM_001126451.1 | XMA-T1R3 | ENSXMAG00000018681 |
| HSA-T1R1 | NM_138697.3 | OLA-T1R1 | NM_001104722.1 |
| HAS-T1R2 | NM_152232.2 | OLA-T1R2a | NM_001104858.1 |
| HSA-T1R3 | NM_152228.1 | OLA-T1R2b | NM_001104723.1 |
| MMU-T1R1 | NM_031867.2 | OLA-T1R2c | NM_001104724.1 |
| MMU-T1R2 | NM_031873.1 | OLA-T1R3 | NM_001104725.1 |
| MMU-T1R3 | NM_031872.2 | GMO-T1R1 | ENSGMOT00000016165 |
| RNO-T1R1 | NM_053305.1 | GMO-T1R2 | ENSGMOT00000017042 |
| RNO-T1R2 | NM_001271266.1 | GMO-T1R3 | ENSGMOT00000002283 |
| RNO-T1R3 | NM_130818.1 | LOC-T1R1 | XM_006642041.1 |
| BTA-T1R1 | XM_005194826.1 | LOC_T1R2-1 | ENSLOCT00000002940 |
| BTA-T1R2 | NM_001206529.1 | LOC_T1R2-2 | ENSLOCT00000006261 |
| CLU-T1R1 | ENSCAFT00000031171 | LOC_T1R2-3 | ENSLOCT00000006278 |
| CLU-T1R2 | NM_001031819.1 | LOC-T1R3 | ENSLOCT00000004773 |
| CLU-T1R3 | NM_001031821.1 | AME-T1R1 | XM_007246328.1 |
| GGA-T1R1 | ENSGALG00000000642 | AME_T1R2-1 | ENSAMXT00000014790 |
| GGA-T1R3 | XM_425740.3 | AME_T1R2-2 | ENSAMXT00000014814 |
| TRU-T1R1 | NM_001097625.1 | AME-T1R3 | ENSAMXT00000003856 |
| TRU-T1R2a | NM_001105217.1 | DRE-T1R1 | NM_001039525.2 |
| TRU-T1R3 | NM_001078629.1 | DRE-T1R2a | AB289805.1 |
| TNI-T1R1 | ENSTNIT00000015161 | DRE-T1R2b | AB289806.1 |
| TNI-T1R2a | ENSTNIT00000015141 | DRE-T1R3 | NM_001039628.1 |
| TNI-T1R2b | ENSTNIT00000015143 | GAC-T1R1 | ENSGACT00000008405 |
| TNI-T1R2b | ENSTNIT00000015144 | GAC_T1R2-1 | ENSGACT00000008620 |
| TNI-T1R3 | ENSTNIT00000018056 | GAC_T1R2-2 | ENSGACT00000008627 |
| ONI-T1R1 | ENSONIT00000002521 | GAC_T1R2-3 | ENSGACT00000008635 |
| ONI-T1R2a | ENSONIT00000002608 | GAC_T1R2-4 | ENSGACT00000008646 |
| ONI-T1R2b | ENSONIT00000002613 | GAC_T1R2-5 | ENSGACT00000008651 |
| ONI-T1R3 | ENSONIT00000005088 | GAC_T1R2-6 | ENSGACT00000008656 |
| XMA-T1R1 | ENSXMAT00000019002 | GAC_T1R2-7 | ENSGACT00000008663 |
| XMA_T1R2-1 | ENSXMAT00000017449 | GAC_T1R2-8 | ENSGACT00000008980 |
| XMA_T1R2-2 | ENSXMAT00000018692 | GAC-T1R3 | ENSGACT00000010071 |

* The abbreviation for species name using the first letter of genus and the first two letters of species in scientific name. DRE-V2RX1 and DRE-V2RH7 were used as the out group.

Supplemental Table S26. Gene conversion analysis for T1R2 gene family of the miiuy croaker and stickleback using coding sequences.

|  | Sequence names | BC KA P-value^a^ | Aligned Offsets | | | Number of  Polymorphisms^b^ | Total  Differences^c^ | Total  Differences^d^ |
| --- | --- | --- | --- | --- | --- | --- | --- | --- |
|  |  |  | Begin | End | Length |  |  |  |
| Miiuy croaker | MIMI-T1R2a;MIMI-T1R2b | 0 | 315 | 864 | 550 | 179 | 17 | 193 |
|  | MIMI-T1R2a;MIMI-T1R2c | 0 | 190 | 1152 | 963 | 279 | 10 | 111 |
|  | MIMI-T1R2b;MIMI-T1R2c | 0 | 321 | 807 | 487 | 167 | 16 | 218 |
|  | MIMI-T1R2b;MIMI-T1R2d | 0.00004 | 401 | 808 | 408 | 135 | 21 | 224 |
|  | MIMI-T1R2c;MIMI-T1R2d | 0 | 691 | 1415 | 725 | 210 | 7 | 116 |
|  | MIMI-T1R2e;MIMI-T1R2f | 0.00036 | 2046 | 2198 | 153 | 29 | 0 | 300 |
|  | GAC_T1R2-1;GAC_T1R2-2 | 0 | 2282 | 2505 | 224 | 55 | 3 | 379 |
| Stickleback | GAC_T1R2-1;GAC_T1R2-2 | 0.00807 | 19 | 75 | 57 | 39 | 6 | 379 |
|  | GAC_T1R2-1;GAC_T1R2-3 | 0 | 1597 | 2444 | 848 | 129 | 21 | 387 |
|  | GAC_T1R2-1;GAC_T1R2-4 | 0 | 2254 | 2444 | 191 | 47 | 5 | 396 |
|  | GAC_T1R2-1;GAC_T1R2-5 | 0.0002 | 2296 | 2444 | 149 | 44 | 7 | 405 |
|  | GAC_T1R2-1;GAC_T1R2-7 | 0.00001 | 2254 | 2444 | 191 | 47 | 7 | 413 |
|  | GAC_T1R2-2;GAC_T1R2-3 | 0 | 2296 | 2468 | 173 | 47 | 4 | 378 |
|  | GAC_T1R2-2;GAC_T1R2-4 | 0.00026 | 1681 | 2151 | 471 | 57 | 9 | 360 |
|  | GAC_T1R2-2;GAC_T1R2-4 | 0.00233 | 2296 | 2468 | 173 | 47 | 7 | 360 |
|  | GAC_T1R2-2;GAC_T1R2-5 | 0.00606 | 1627 | 2154 | 528 | 59 | 12 | 368 |
|  | GAC_T1R2-3;GAC_T1R2-4 | 0.00082 | 78 | 1772 | 1695 | 517 | 45 | 113 |
|  | GAC_T1R2-3;GAC_T1R2-5 | 0.00018 | 74 | 1679 | 1606 | 511 | 38 | 108 |
|  | GAC_T1R2-3;GAC_T1R2-6 | 0 | 74 | 1594 | 1521 | 507 | 38 | 145 |
|  | GAC_T1R2-3;GAC_T1R2-7 | 0.00021 | 78 | 1772 | 1695 | 517 | 43 | 113 |
|  | GAC_T1R2-3;GAC_T1R2-8 | 0 | 76 | 1568 | 1493 | 496 | 48 | 153 |
|  | GAC_T1R2-4;GAC_T1R2-6 | 0.00039 | 19 | 1588 | 1570 | 542 | 39 | 104 |
|  | GAC_T1R2-4;GAC_T1R2-8 | 0 | 78 | 1454 | 1377 | 461 | 34 | 135 |
|  | GAC_T1R2-5;GAC_T1R2-6 | 0.00001 | 21 | 1594 | 1574 | 542 | 38 | 110 |
|  | GAC_T1R2-5;GAC_T1R2-8 | 0 | 76 | 1568 | 1493 | 496 | 38 | 140 |
|  | GAC_T1R2-6;GAC_T1R2-7 | 0 | 78 | 1594 | 1517 | 503 | 39 | 138 |
|  | GAC_T1R2-6;GAC_T1R2-8 | 0 | 76 | 1458 | 1383 | 465 | 35 | 135 |
|  | GAC_T1R2-7;GAC_T1R2-8 | 0 | 78 | 1568 | 1491 | 494 | 46 | 143 |

a Bonferroni-corrected KA (BLAST-like) P values.

b The number of polymorphic sites within gene-converted fragments of each group.

c The number of mismatched sites between gene-converted paralogous fragments from the same species.

d Total number of mismatched sites between aligned paralogous sequences from the same species, respectively.

## Supplemental Table S27. The number of OR functional genes belonging to different groups in some species.

|  | **Type1** |  |  |  |  |  | **Type2** |  |
| --- | --- | --- | --- | --- | --- | --- | --- | --- |
| **Species** | **Air** | **Air+Water** | **Air** | **Water** | **Water** | **Water** | **Water** | **Total number** |
|  | **α** | **β** | **γ** | **δ** | **ε** | **ζ** | **η** |  |
| Miiuy croaker  Fugu  Tetraodon  Stickleback  Zebrafish  Human | 0  0  0  0  0  58 | 1  1  0  1  4  329 | 0  0  0  0  1  0 | 61  30  4  71  62  0 | 1  2  2  4  12  0 | 7  4  2  18  37  0 | 43  10  3  8  38  0 | 113  47  11  102  154  387 |

## Supplemental Table S28. Vomeronasal receptor gene repertoire in vertebrates.

| **Species** | **V1R** | **V2R** |
| --- | --- | --- |
| Zebrafish | 2 | 40 |
| Fugu | 1 | 18 |
| Tetraodon | 1 | 12 |
| Stickleback | 1 | 24 |
| Miiuy croker | 6 | 40 |
| Frog | 22 | 248 |
| Mouse | 211 | 121 |
| Human | 4 | 0 |

# Supplemental Notes

## 1. *De novo* sequencing and assembly of the miiuy croaker genome.

***1.1 Library preparation and data generation.***

The whole genome shotgun strategy and next-generation sequencing technologies on the Illumina HiSeq 2000 sequencing platform were used to sequence the genome of the miiuy croaker. Genomic DNA was extracted from a wild female miiuy croaker from the East China Sea area of Zhejiang Province using Pure gene Tissue Core Kit A (Qiagen). We constructed 7 pair-end libraries with short insert size from 180 bp to 800 bp and 4 mate-pair libraries with long insert size 3 kb, 8 kb and 20 kb according to the Illumina standard protocol, and read lengths were 101 bp or 151 bp. Finally, 136.29 Gb of raw data were generated, and 100.79 Gb was retained for assembly after filtering out low-quality and duplicated reads (Table S1).

***1.2 Genome size estimation of the miiuy croaker.***

The genome size of the miiuy croaker was firstly estimated by flow cytometry analysis using human erythrocytes as an internal standard. The DNA content of miiuy croaker was estimated to be 0.67 pg, corresponding to 655.26 Mb (Figure S2). In order to give an accurate estimate of the genome size, we employed K-mer depth distribution of sequence reads to estimate the genome size^61^. In other words, genome size can be estimated if the amount of sequencing data and the depth are known. The genome size, G, can be calculate from the formula G=K-mer number/K-mer depth, where the K-mer number is the total number of K-mers, and K-mer depth is the peak value of the frequency curve overall sequencing depth. Here, we generated 76.52 Gb of reads from the short insert size libraries, 21-mers were extracted from these reads. Finally, in the present study, K-mer number is 64,258,331,034 and K-mer depth is 101, we therefore estimated the genome size to 636.22 Mb (Figure S1 and Table S2), which is close to the 655.26 Mb estimated by flow cytometry analysis.

***1.3 De novo assembly.***

The genome was *de novo* assembled by the software program Allpaths-LG^9^, based on the *de bruijn* graph theory. We used the reads from the 7 short insert size pair-end libraries (180-800 bp) to assemble the contigs. Reads from the 4 mate-pair libraries with insert size 3 kb, 8 kb and 20 kb were aligned onto the contigs for the scaffolds building using paired-end information. Then, the paired-end information was used to link contigs to scaffolds ranging from short insert size to long insert size, step by step. The gaps in the scaffolds were filled using the package Gapcloser^34^ (version 1.12, http://sourceforge.net/projects/soapdenovo2/files/GapCloser/). Consequently, the assembly produced a genome of 619.30 Mb with a contig N50 of 73.32 kb and a scaffold N50 of 1.15 Mb (Table 1 and Table S3,S4).

***1.4 Evaluation of the accuracy of the genome assembly.***

Soapligner^10^ was used to realign all usable reads onto the assembled scaffolds to validate the single-base accuracy of the genome assembly. The peak sequencing depth was 127-fold and 92.79% of the genome assembly was more than 50-fold (Figure S3). Then, the genomic coverage was evaluated by aligning the publicly available 3,221 expressed sequence tags (ESTs)^3^ and 69,071 transcriptome unigenes^4^ to the assembly using BLAT^11^. The genome assembly covered 95.81% and 95.59% of the ESTs and unigenes, and 92.86% and 85.94% of the covered high-quality EST and unigenes (length≥1000bp) exist in one scaffold, making sure that the assembly covered most of the transcriptional region (Table S5). The GC content in the miiuy croaker genome was calculated as the percentage of G and C in the non-N nucleotides to analyze nucleotide distribution and check the randomness of sequencing. The miiuy croaker genome has a GC content of 40.96%, which is slightly higher than zebrafish and medaka, but lower than the other sequenced teleosts. As the GC contents of zebrafish (36.6%), medaka (40.1%), stickleback (44.6%), tetraodon (46.4%) and fugu (45.5%) genomes were also calculated for comparison purpose (Figure S4). We also compared the assembled genome with other fish genomes that displayed the similarly size and composition. All of these results indicated the high quality assembly of the miiuy croaker genome (Table S4).

## 2. Genomic features

***2.1 Repeat annotation.***

After the accurate assembly of the miiuy croaker genome, we performed the repeat annotation. There are two main types of repeats in the genome, tandem repeats and transposable elements. Tandem Repeat Finder (TRF) (http://tandem.bu.edu/trf/trf.html, version 4.04) with parameters “Match=2, Mismatch=7, Delta=7, PM=80, PI=10, Minscore=50 and MaxPeriod=12”^35^ and RepeatMasker were used to identify the tandem repeats, including the simple sequence repeat, satellites and low complexity repeats^36^. We identified the transposable elements by homology search and *de novo* prediction. For the former, RepeatMasker (http://www.repeatmasker.org, version 3.3.0) and RepeatProteinMask (http://www.repeatmasker.org/, version 3.2.2) were carried out against RepBase TE library and TE protein library, respectively. For the latter, RepeatModeler (http://www.repeatmasker.org/RepeatModeler.html, version 1.05, default parameter) was used to generate a *de novo* TE library.

***2.2 Identification of non-coding RNA genes.***

The transfer RNAs (tRNAs) in genomic sequence were predicted by tRNAscan-SE with eukaryote parameters^37^. BLASTN^38^ was used to identify the ribosomal RNAs (rRNAs) by aligning the eukarya rRNA sequences from the SILVA database^39^. For microRNAs (miRNAs) and snRNA identification, INFERNAL^62^ was used by searching against the Rfam database^63^ with default parameters. Finally, 1,824 non-coding RNA, including 73 rRNA, 522 miRNA and 1,229 tRNA genes were identified (Table S16,S17).

***2.3 Detection of Single nucleotide polymorphism (SNP) and InDel.***

To detect the SNPs and InDels in the miiuy genome, we used Soapligner^4^ to align high-quality reads from short insert size to the assembly with at “local” condition and other parameters being default. Then, the package SOAPsnp^64^ was preformed SNP calling, and finally obtained 1,387,371 single-nucleotide polymorphisms (SNPs) including, which represents a heterozygous rate in the miiuy croaker of 2.24×10^-3^. Additionally, we performed InDel calling for the miiuy croaker genome using a Bayesian approach implemented in the package SAMtools^65^ with a total of 382,008 InDels were identified.

***2.4 Gene prediction.***

The genes in the miiuy croaker were predicted with a method integrating the homology-based, transcriptome-based and *ab initio* prediction. For the homology-based prediction, the proteins of *Danio rerio* (zebrafish), *Tetraodon nigroviridis* (tetraodon), *Takifugu rubripes* (fugu), *Xiphophorus maculates* (platyfish), *Gasterosteus aculeatus* (stickleback), *Oryzias latipes* (medaka), *Homo sapiens* (human) and *Mus musculus* (mouse) were downloaded from Ensembl (version 77) and mapped onto the genome using TBLASTN with E value<1E-5. Then, homologous genome sequences were aligned against the matching proteins using GeneWise to generate gene model structures^41^. Transcriptome reads of the miiuy croaker^4^ were aligned to genomic sequences by Tophat^42^, and transcript structures were obtained using Cufflinks^43^. *Ab initio* prediction was performed by Augustus^44^, GlimmerHMM^45^ and SNAP^46^ with appropriate parameters to predict the protein-coding genes. The final gene model was generated by integrating all above gene sets using Glean^17^, containing a total of 21,960 protein coding genes.

***2.5 Functional annotation of genes.***

Functional annotation of the predicted genes were assigned according to the best match of the alignment to a number of nucleotide and protein sequence databases, including Nt, Nr, SwissProt, KOG, and InterPro with BLAST searches using the E-value cutoff of 1E-5. We used NCBI CDD and InterProScan (Pfam, PRINTS, PROSITE, ProDom, and SMART databases)^47^ to determine motifs and domains in the final gene set. Gene ontology (GO) functional classification for these annotated genes was performed using the annotation retrieved from Nr by Blast2GO. A total of 15,413 genes were classified into at least one functional categorie under the three ontologies of GO (biological process, cellular component, and molecular function) (Figure S7). We also mapped the miiuy croaker protein-coding genes to metabolic pathways and identified the best match for each gene using KAAS^50^ based on the KEGG database. Altogether, 11,181 protein coding genes were assigned to 342 KEGG pathways summarizing into 40 subgroups, as the signal transduction was the dominated group (1,873), followed by cancer (1,462), infectious diseases(1,314), endocrine system (738) and immune system (729) (Figure S8).

## 3. Comparative genomics and evolution

***3.1 Gene family cluster.***

To define gene families among 21 chordate genomes, the protein-coding genes from 19 species were downloaded from Ensembl version 77 (October 2014) human, mouse, cow, chicken, lizard, xenopus, amazonmolly, cavefish, cod, fugu, medaka, platyfish, spotted gar, stickleback, tetraodon, tilapia, zebrafish, coelacanth, lamprey and *C. intestinalis*. The elephant shark dataset was downloaded from Elephant shark genome Project (<http://esharkgenome.imcb.a-star.edu.sg/>)^66^ and the miiuy croaker proteome is from the present study. The longest transcript isoform were selected to represent each gene, and the protein sequence less than 30 amino acids were filtered out. We used OrthoMCL 2.0.9^51^ to define gene families descended from a single gene in the common ancestor, and BLASTP to determine the similarities between genes in three with an e-value cutoff of 1E-5. We obtained 24,093 gene families and 560 single-copy gene families.

***3.2 Gene familyexpansion and contraction.***

Expansion and contraction of gene family analysis was processed using CAFE 3.1^52^ to identify the evolutionary dynamics of gene families as a stochastic birth and death process. The gene families of the miiuy croaker were compared with those of other genomes (fugu, tetraodon, stickleback, medaka, zebrafish, human, spotted gar, elephant shark and coelacanth), and families with conditional *P*-values less than threshold (0.05) were determined as expansion and contraction.

***3.3 Phylogenetic analysis and divergence time estimation.***

We constructed a phylogenetic tree of the miiuy croaker with other 20 chordate genomes (human, mouse, cow, chicken, lizard, xenopus, amazonmolly, cavefish, cod, fugu, medaka, platyfish, spotted gar, stickleback, tetraodon, tilapia, zebrafish, coelacanth, lamprey, *C. intestinalis* and elephant shark). The resulting 560 single-copy orthologous gene families were aligned by MAFFT 7.205^53^ and concatenated to one super protein sequence for each species. Gblocks 0.91b^54^ was used to trim the concatenated alignment and ProtTest 3.4^55^ was used to determine the best-fit model (JTT+I+G+F). The phylogenetic tree was reconstructed from the 560 single-copy gene families both using RAxML 8.1.5^56^ and MrBayes 3.2^57^. The divergence time between species were estimated using MCMCTREE program implemented in the PAML. To further confirm the relationship between miiuy croaker and stickleback, pairwise comparison of protein sequences of five representive chordates species was carried on. The comparison revealed the identity between miiuy croaker and stickleback was higher than miiuy croaker to other species, which supplied robust supporting for our genome-level phylogeny (Figure S11). Summarizing, the topological structure of the phylogenetic tree and higher identity of orthologous proteins using genomic level data set indicate a closer affinity of Gasterosteiformes and Sciaenidaes.

***3.4 Gene Ontology functional enrichment***

Gene ontology (GO) enrichment was performed by Blast2GO^48^. The whole-annotated gene set was mapped to GO terms in the database (http://www.geneontology.org/) to use as a background. Then, significantly enriched GO terms in target genes were identified by comparing to the GO annotations of the whole genome. A Fisher's exact test was used to correct for multiple testing with statistical significance at 0.05.

## 4. Sensory adaptation to habitats

***4.1 Visual modification.***

To identify the vision pigment genes in the miiuy croaker, we extracted the closely related species from Genbank (Table S23) as query sequences to search the miiuy croaker genome by BLASTN and TBLASN with E-value<1E-10 and obtained the scaffold that vision pigment genes located in. The coding sequences were ensured by MAFFT compared with the closely related species. Finally, we identified four types of vision pigment genes (RH1, RH2, SWS2 and LWS) and found SWS1 was lost in the miiuy croaker. Furthermore, to further confirm these vision pigment genes, we analyzed several conserved genes that existed in the upstream and downstream of visual opsin genes. The locations and genomic synteny surrounding of all opsin genes were showed in Figure S12.

The amino acid sites involved in the light sensitivities are crucial for spectral tuning, we compared the amino acid sequences of RH1, RH2, SWS2 and LWS in the miiuy croaker with six other teleosts (Figure S13). We surveyed opsin genes of the vertebrate species to reconstruct the evolutionary history of opsin genes and proposed the evolutionary pattern of RH1 and RH2 (Figure S14).

***4.2 T1R2 expansion in the miiuy croaker.***

Taste receptor (TR) genes were searched in themiiuy croaker genome by BLASTN and TBLASN with E-value<1E-10 against the genomic data, using annotations of TR genes as queries (Table S25). The target regions were selected to predict the coding sequences using GeneWise based on homology prediction method. The coding sequences were re-checked against the NCBI database by BLAST searches for further confirm. Furthermore, the coding sequences were used to predict the transmembrane (TM) domains by TMHMM Server v. 2.0. If a predicted gene with frameshifts, disrupting stop codons, or incomplete regions from TM1 to TM7, it was regarded as a pseudogene. Phylogenetic trees were constructed by the Bayesian approach. Synteny was analysed using the Genomicus^59^, Ensemble database and Map Viewer in NCBI. Number data were from Ensemble database and previous reports^32,67,68^. Gene conversion analysis was performed by GENECONV (version 1.8)^60^ with 10,000 pseudo-replicates and one mismatch allowed (p < 0.01).

***4.3 Olfactory receptor (OR) and vomeronasal receptor (V1R and V2R) genes in the miiuy croaker.***

We extracted the representative OR and VR sequences as queries to search Olfactory receptor (OR) and vomeronasal receptor (V1R and V2R) genes in the miiuy croaker (Table S27,S28). Miiuy croaker OR and V1R/V2R proteins that we obtained were re-cheaked by BLAST in NCBI to further confirm these genes. Neighbor-joining (NJ) phylogenetic tree was constructed (Figure S15) using functional OR genes of amphioxus (31), zebrafish (154), large yellow croaker (28), miiuy croaker (113) and six non-OR GPCRs in human as outgroup. These sequences of amphioxus and zebrafish in this study were obtained from the datasets of a previous study^69^ and a part of large yellow croaker sequences came from a research article^70^. These sequences of outgroup were extracted from NCBI (NP_000670.1, NP_000729.2, NP_001044.1, NP_001287.2, NP_005292.2 and NP_037477.1). The number of VRs was obtained from CRDB (<http://zldev.ccbr.utoronto.ca/CRDB/statistics.jsp>) and a previous study^71^.

# Supplemental References

1. Huang, S. *et al*. The genome of the cucumber, *Cucumis sativus* L. *Nat. Genet.* **41**, 1275-1281 (2009).
2. Nawrocki, E. P., Kolbe, D. L. & Eddy, S. R. Infernal 1.0: inference of RNA alignments. *Bioinformatics* **25**, 1335-1337 (2009).
3. Griffiths-Jones, S. *et al*. Rfam: annotating non-coding RNAs in complete genomes. *Nucleic Acids Res*. **33**, D121-D124 (2005).
4. Li, R. *et al*. SNP detection for massively parallel whole-genome resequencing. *Genome Res*. **19**, 1124-1132 (2009).
5. Li, H. *et al*. The Sequence Alignment/Map format and SAMtools. *Bioinformatics* **25**, :2078-2079 (2009).
6. Venkatesh, B. *et al*. Survey sequencing and comparative analysis of the elephant shark (*Callorhinchus milii*) genome. *PLoS Biol*. **5**, e101 (2007).
7. [Ishimaru, Y](http://www.ncbi.nlm.nih.gov/pubmed/?term=Ishimaru Y%5BAuthor%5D&cauthor=true&cauthor_uid=16274966). *et al*. Two families of candidate taste receptors in fishes. *Mech. Develop*. **122**, 1310-1321 (2005).
8. Shi, P. & Zhang, J. Contrasting modes of evolution between vertebrate sweet/umami receptor genes and bitter receptor genes. *Mol. Biol. Evol.* **23**, 292-300 (2006).
9. Niimura Y. On the origin and evolution of vertebrate olfactory receptor genes: comparative genome analysis among 23 chordate species. *Genome Biol.* Evol. **1**, 34-44 (2009).
10. Zhou, Y. *et al*. Family structure and phylogenetic analysis of odorant receptor genes in the large yellow croaker (*Larimichthys crocea*). *BMC Evol. Biol.* **1**, 237 (2011).
11. Shi, P. & Zhang, J. Comparative genomic analysis identifies an evolutionary shift of vomeronasal receptor gene repertoires in the vertebrate transition from water to land. *Genome Res.* **17**, 166-174 (2007).
